# Supplementary material for: Bead-jet printing enabled sparse mesenchymal stem cell patterning augments skeletal muscle and hair follicle regeneration
Source: Nat Commun. 2022 Dec 3;13:7463. doi: 10.1038/s41467-022-35183-8 (PMC9718784; doi:10.1038/s41467-022-35183-8)
Supplement: Supplementary file 1 — Supplementary Information [file 41467_2022_35183_MOESM1_ESM.pdf]

Supporting information for

Bead-jet printing enabled sparse MSCs patterning augments skeletal muscle and hair follicle regeneration

Yuanxiong Cao[1,2,#], Jiayi Tan[1,2,#], Haoran Zhao[1,2,#], Ting Deng[1,2,#], Yunxia Hu[1,2], Junhong Zeng[1,2], Jiawei Li[1,2], Yifan Cheng[1,2], Jiyuan Tang[1,2], Zhiwei Hu[1,2], Keer Hu[1,2], Bing Xu[1,2,3], Zitian Wang[1,2], Yaojiong Wu[1,2], Peter E. Lobie[1,2,3], Shaohua Ma[1,2,3,4,*]

[1]Tsinghua Shenzhen International Graduate School (SIGS), Tsinghua University, Shenzhen, 518055, China

[2]Tsinghua-Berkeley Shenzhen Institute (TBSI), Shenzhen, 518055, China

[3]Shenzhen Bay Laboratory, Shenzhen, 518055, China

[4]Institute for Brain and Cognitive Sciences, Tsinghua University, Beijing, 100084, China

[#]These authors contributed equally

[*]Correspondence: ma.shaohua@sz.tsinghua.edu.cn (S.M.)

This file contains 50 figures, 2 tables and 6 videos.

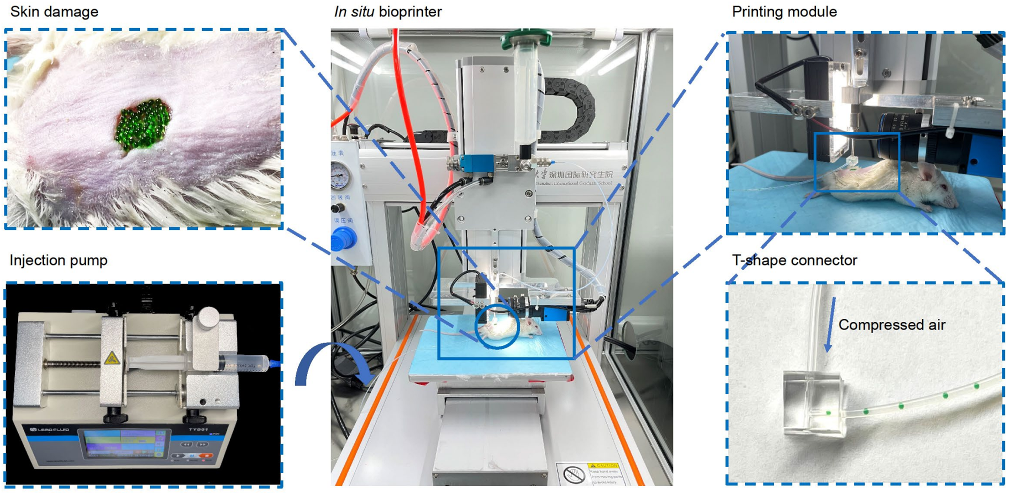

Figure S1. The bead-jet printing setup.

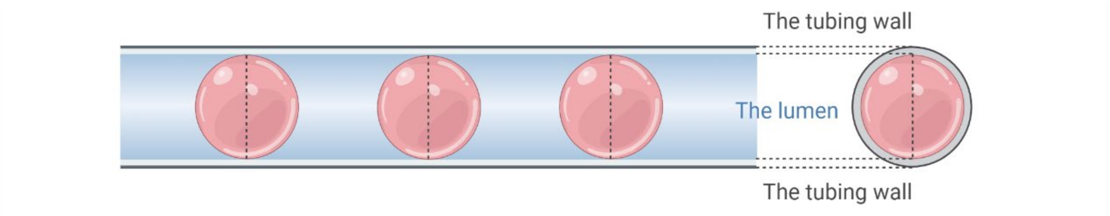

Figure S2. Schematic of the surfactant-free droplet production. Gel droplets in PTFE tubing, from the side view and the transverse view, respectively. The droplet occupies the entire cross-section of the tubing lumen, preventing droplet-droplet collision and fusion.

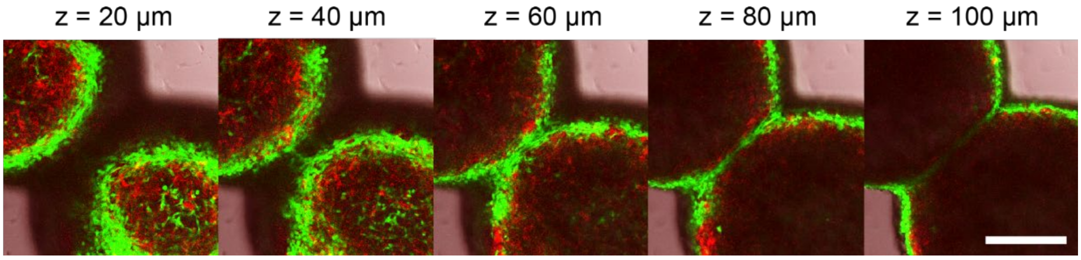

Figure S3. Zoom-in images of spheroids merging at day 2 after assembly. The spheroids were fabricated from Matrigel suspended with 1.5 × 10^7 mL^{-1} NIH3T3/GFP and 1.5 × 10^7 mL^{-1} OVCAR-5/RFP. Scale bar, 250 µm.

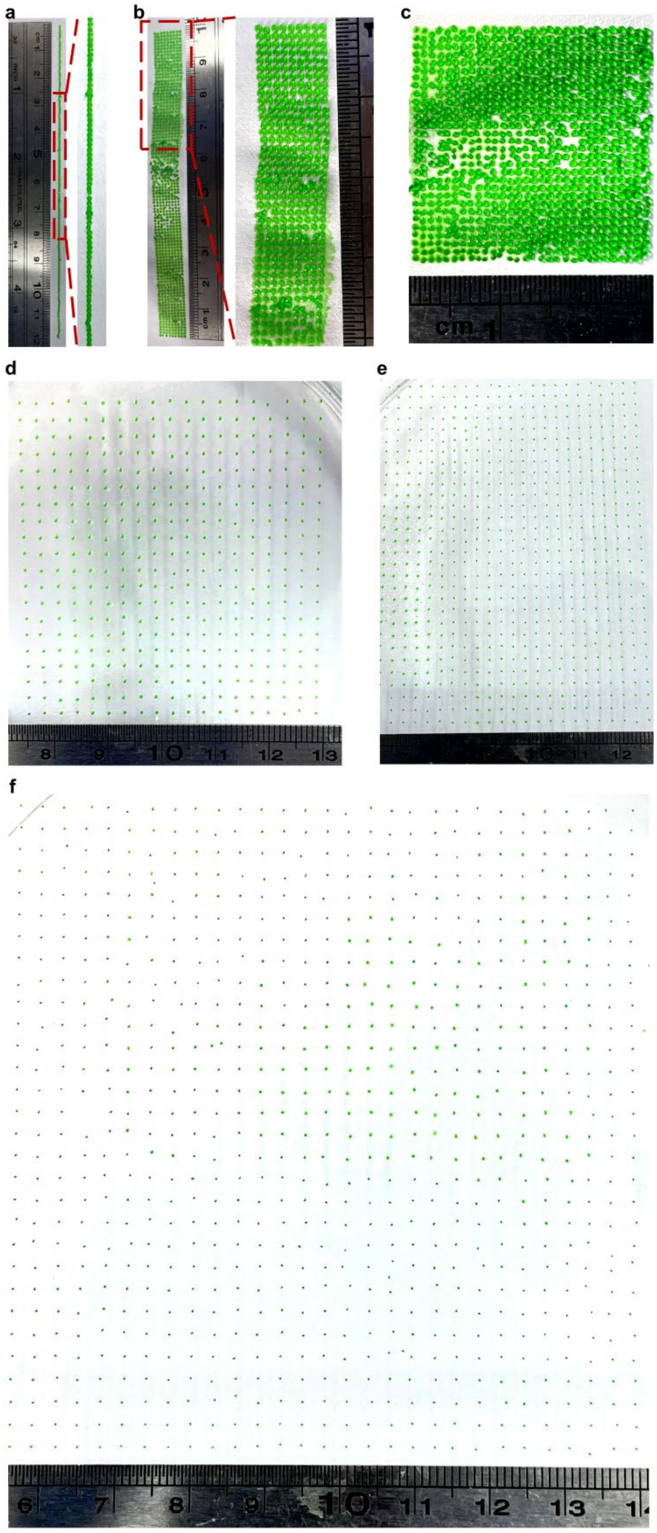

Figure S4. Printing of microbeads into (**a**) a 12-cm long straight line, (**b**) a rectangular shape (10 cm × 1 cm), (**c**) a square shape (3 cm × 3 cm), and (**d-f**) large-scale shapes (**d**, 5 cm × 7 cm; **e**, 8 cm × 12 cm; **f**, 9 cm × 9 cm).

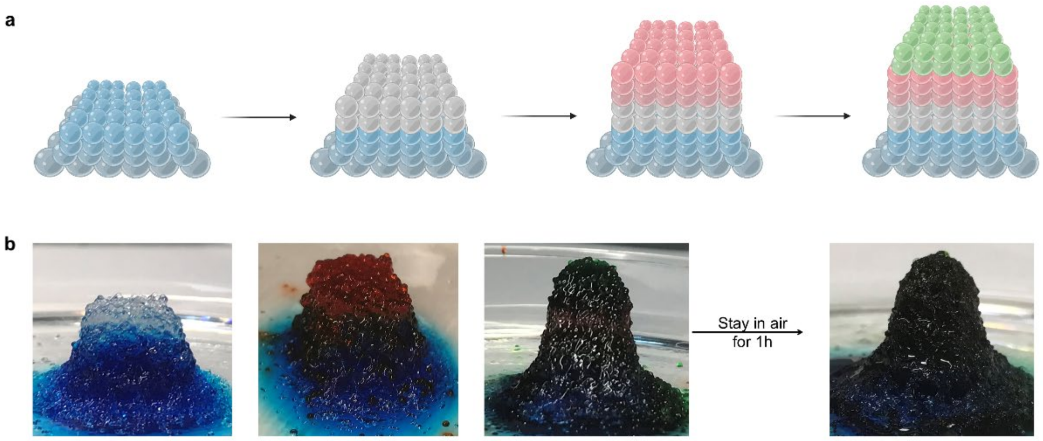

Figure S5. Printing of 3D heterogeneous structures. a, Schematic of heterogeneous printing. **b**, 3D printed heterogeneous structures of gelatin beads on sacrificial gelatin support. The printed assembly had up to 50 layers. Scale bar, 1 cm.

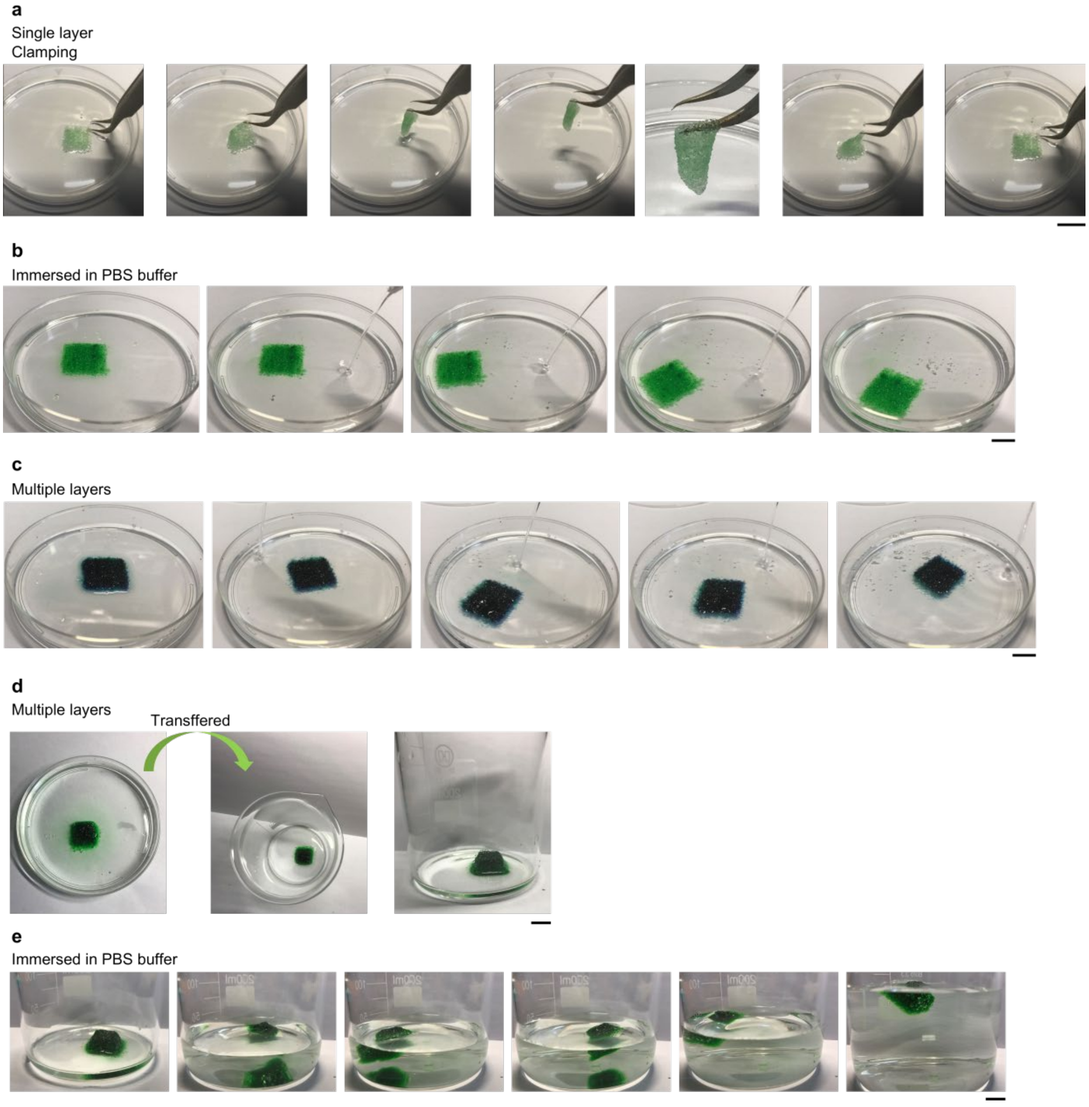

Figure S6. Evaluation of structural integrity of printed architectures. a, Clamping of a planar structure (single layer). **b**, A planar structure (single layer) immersed in PBS buffer. **c**, A planar structure (multiple layers) immersed in PBS buffer. **d**, A 3D cube structure transferred from a 10-cm culture dish to a beaker. **e**, A 3D cube structure immersed in PBS buffer in a beaker. Scale bar, 1cm.

a

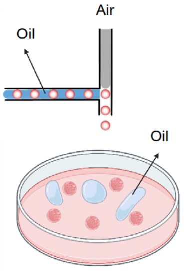

Parameter	HFE 7000	HFE 7100
Boiling point (°C)	34	61
Molecular Weight (g/mol)	200	250
Liquid Density (g/ml)	1.4	1.52
Surface Tension (dynes/cm)	12.4	13.6

b

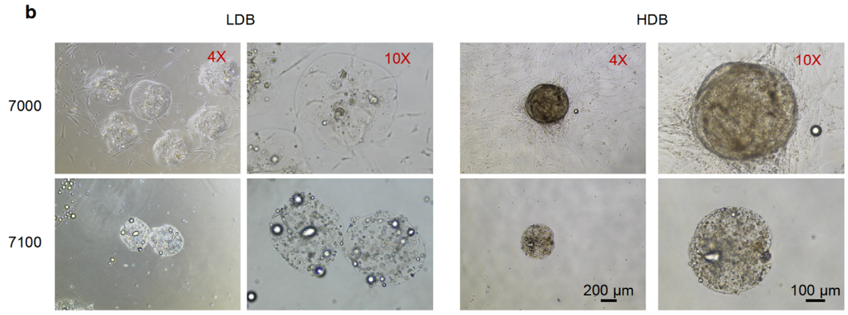

Figure S7. Effects of oil residues on MSCs growth in the Matrigel LDB and HDB groups. The oil was dispersed in the tubing during droplet formation and then ejected into the cell culture media during printing by compressed air. **a**, The parameters of HFE 7000 and HFE 7100 oil. **b**, MSCs cultured in the Matrigel LDB and HDB groups with 7000 and 7100 ejection for 7 days. HFE7000 was used throughout the study. Schematics were created with biorender.com.

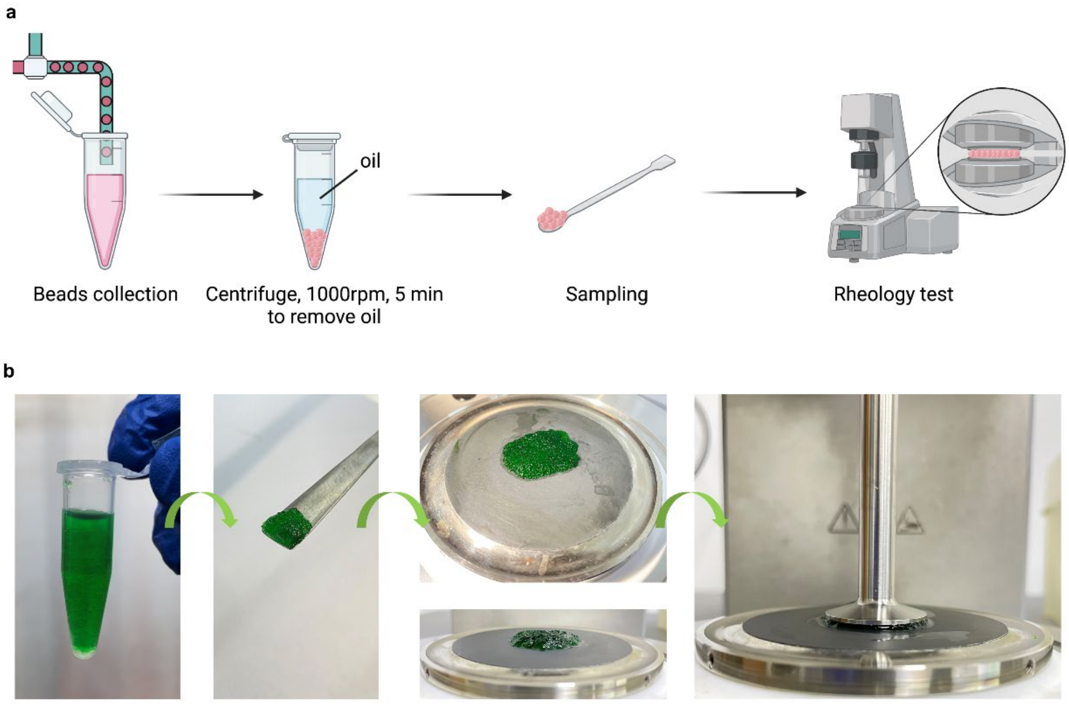

Figure S8. Procedures of the rheological test of microbead assembly. a, Schematic of microbead assembly preparation for the rheological test. **b**, Experimental procedures of the rheological test of microbead assembly. Schematics were created with biorender.com.

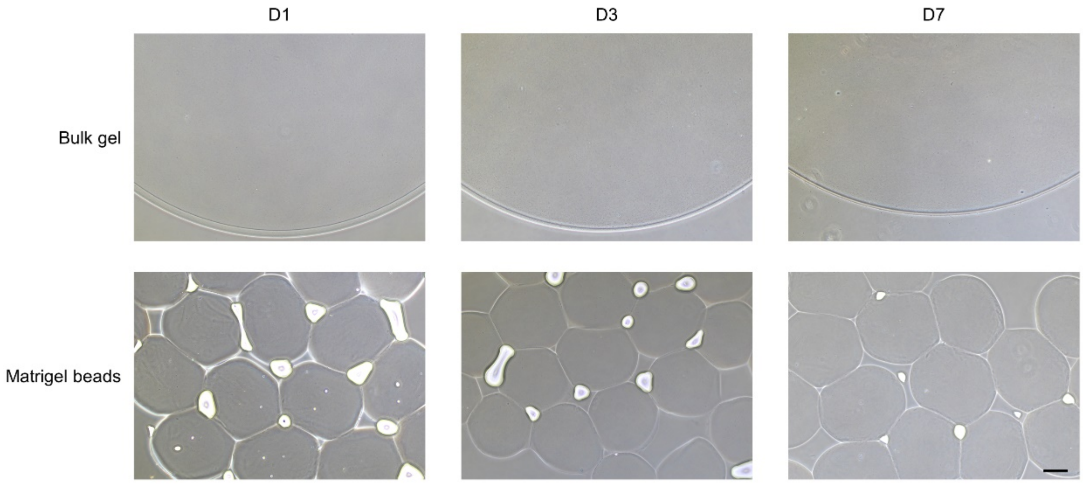

Figure S9. Degradation test of bulk Matrigel and acellular Matrigel beads *in vitro* at D1, D3 and D7. Scale bar, 200 μm.

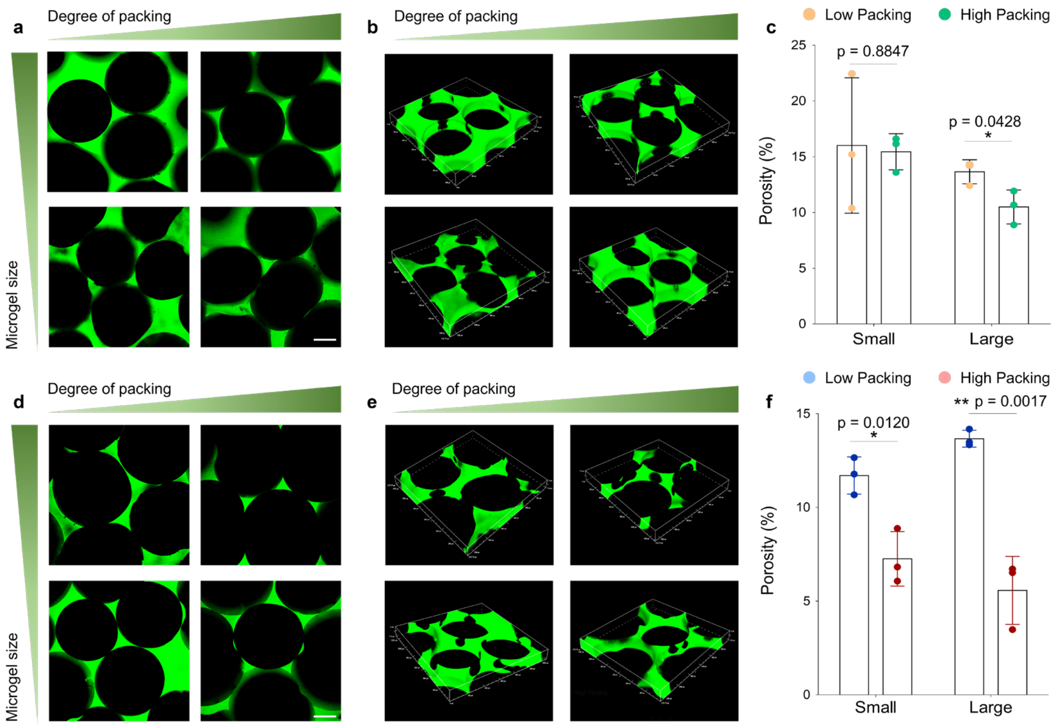

Figure S10. Porosity characterization of granular GelMA and HAMA hydrogels. a-c, Representative confocal 2D (**a**) and 3D (**b**) representative images of granular GelMA hydrogels assembled from different microbeads (D = 450 μm and 650 μm) and packing densities. The bead assemblies were infiltrated with FITC-dextran solution. (**c**) Quantification of granular GelMA porosity. n = 3. **d-f**, Representative confocal 2D (**d**) and 3D (**e**) representative images of granular HAMA hydrogels assembled from different microbeads (D = 450 μm and 650 μm) and packing densities. The bead assemblies were infiltrated with FITC-dextran solution. (**f**) Quantification of granular HAMA porosity. n = 3. Here, the high-packing and low-packing conditions were reached by centrifugation of the microbead assembly for 5 min at 1000 rpm or at 15, 000 rpm. Scale bar, 200 μm. The data is represented as mean ± SD. *p < 0.05; **p < 0.01; Significant difference is calculated by unpaired two-failed student t-test.

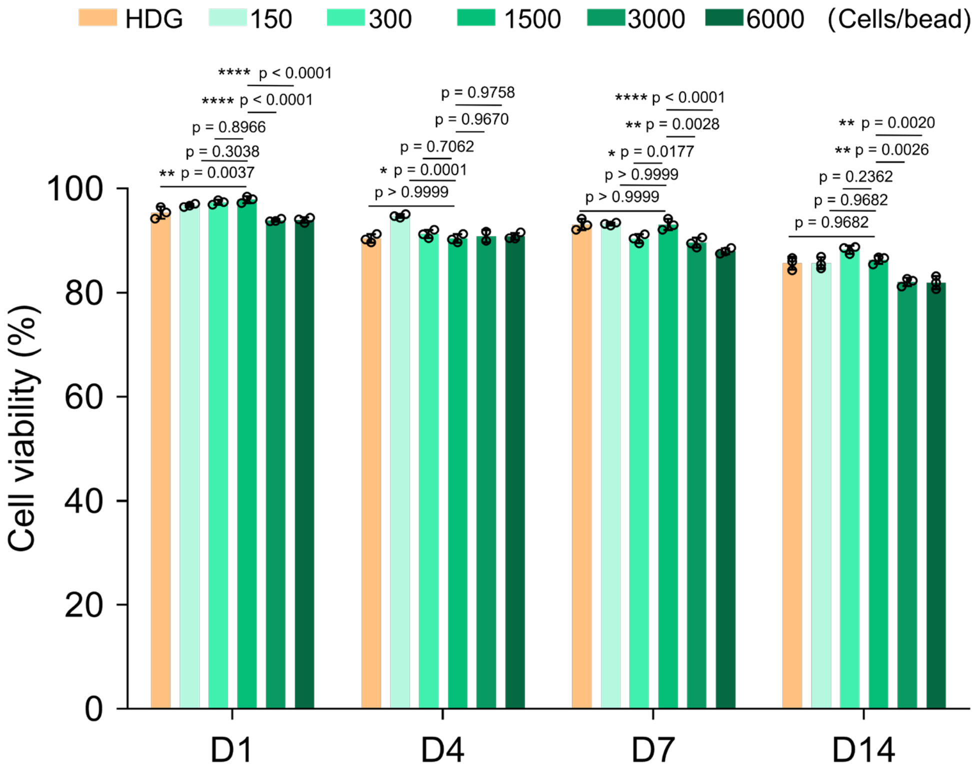

Figure S11. Quantification of MSCs viability in the Matrigel HDG, and Matrigel beads comprising different MSCs densities at D1, D4, D7 and D14. n = 3 for each group. Hydrogel samples were from 3 different batches. For each batch, three random imaging section were chosen and the average value was used to plot the data. The data is represented as mean ± SD. Significant difference is determined by one-way ANOVA, followed by Tukey's test. *p < 0.05; **p < 0.01; ***p < 0.001; ****p < 0.0001.

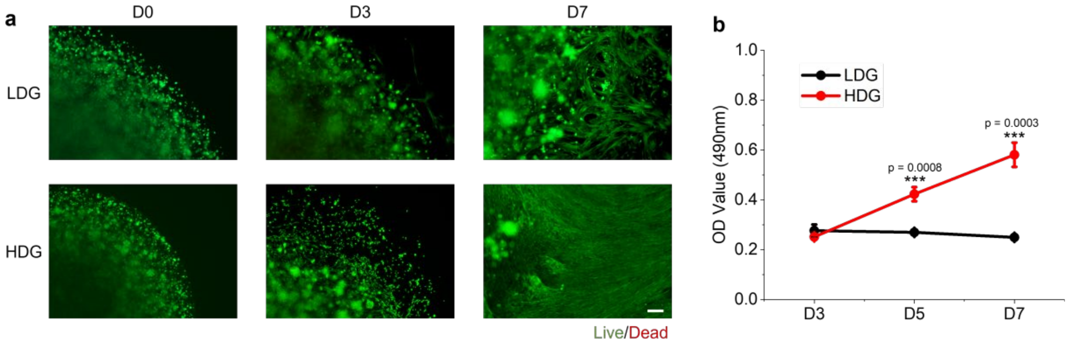

Figure S12. Characterization of MSC viability and growth in the Matrigel LDG and HDG groups. a, MSCs were cultured in the Matrigel LDG and HDG groups and imaged at D0, D3, and D7. Live cells were stained with Calcein-AM (green) and dead cells were stained with propidium iodide (PI, red). Scale bar, 200 µm. **b**, OD values at 490 nm of proliferated MSCs cultured in the Matrigel LDG and HDG groups at D3, D5 and D7. n = 3. The data is represented as mean ± SD. ***p < 0.001; Significant difference is calculated by unpaired two-failed student t-test.

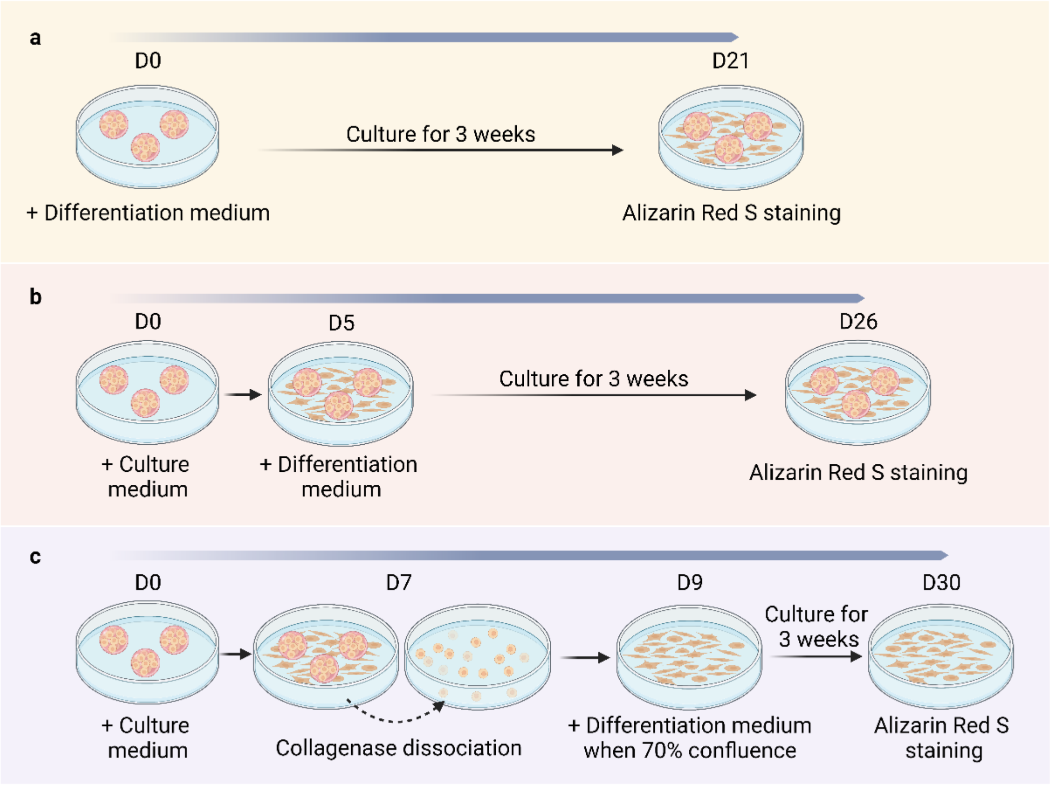

Figure S13. Schematic illustration of osteogenic differentiation of MSCs-Matrigel complexes cultured at different conditions. a, Direct differentiation of microbeads from D0. **b**, Differentiation of microbeads from D5. The microbeads were cultured using normal cell culture medium (DMEM / F12 supplemented with 10% FBS and 1% penicillin/streptomycin) for 5 days before transferring to differentiation medium. **c**, Differentiation of MSCs retrieved from microbeads after being cultured in normal cell culture medium for 7 days. All differentiation tests were tracked for 3 weeks. Schematics were created with biorender.com.

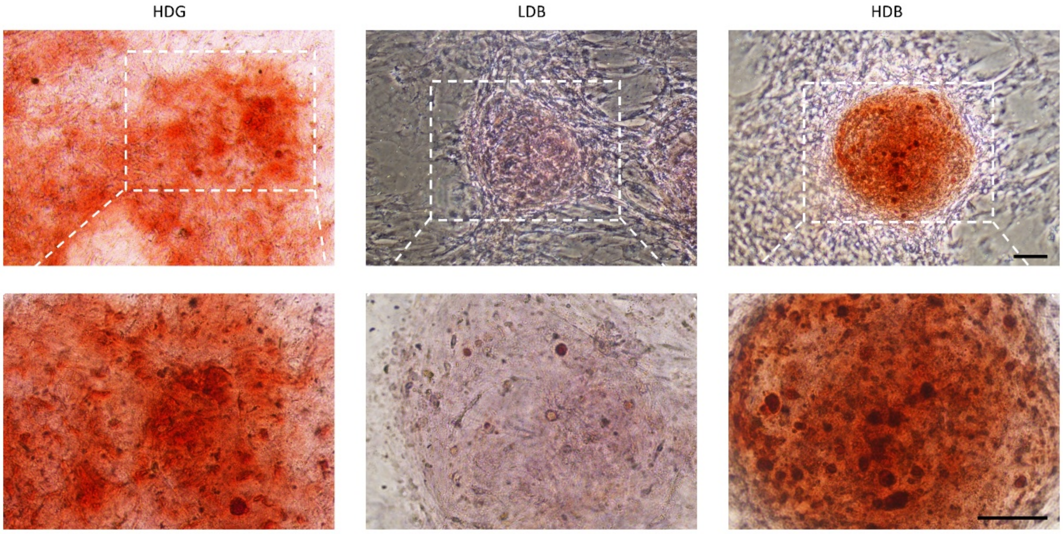

Figure S14. Osteogenic differentiation of microbeads from D5. The microbeads were cultured using normal cell culture medium for 5 days and then the culture medium was replaced using the osteogenic differentiation medium. Scale bars, 100 µm.

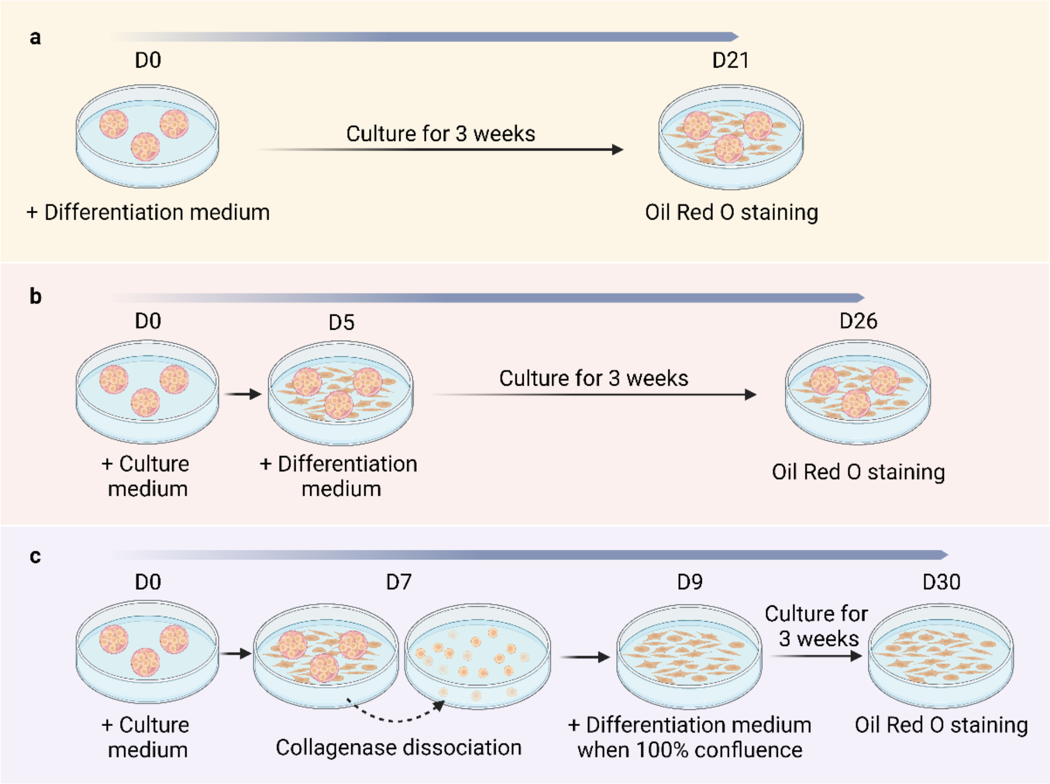

Figure S15. Schematic illustration of adipogenic differentiation of MSCs-Matrigel complexes cultured at different conditions. **a**, Direct differentiation of microbeads from D0. **b**, Differentiation of microbeads from D5. The microbeads were cultured using the normal cell culture medium for 5 days. **c**, Differentiation of MSCs retrieved from microbeads after being cultured in normal cell culture medium for 7 days. All differentiation tests were tracked for 3 weeks. Schematics were created with biorender.com.

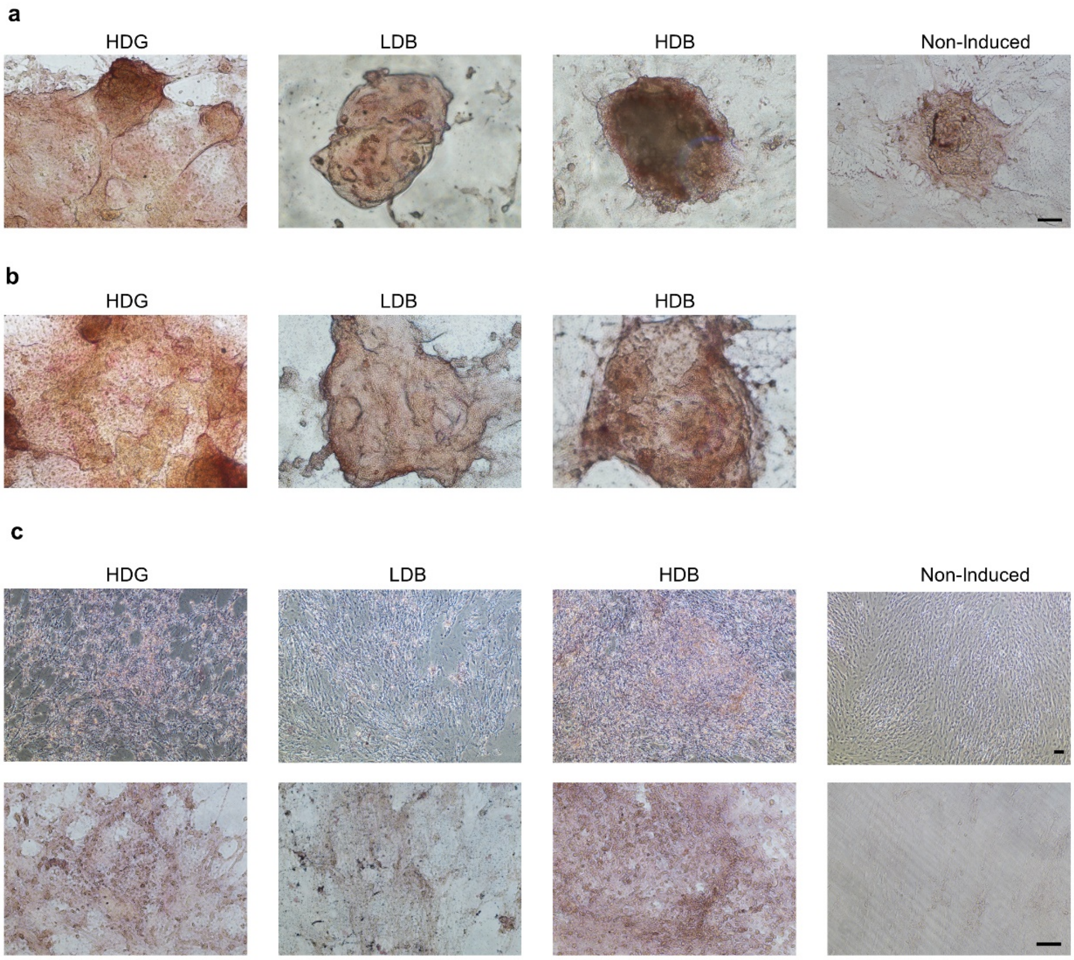

Figure S16. Adipogenic differentiation of MSCs-Matrigel complexes cultured at different conditions. a, Adipogenic differentiation of microbeads from D0. **b**, Adipogenic differentiation of microbeads from D5. The microspheres were cultured using normal cell culture medium for 5 days and then the culture medium was replaced using adipogenic differentiation medium. **c**, Adipogenic differentiation of MSCs retrieved from microbeads after being cultured in normal cell culture medium for 7 days. All differentiation lasts for 3 weeks and stained using Oil Red O staining. Scale bars, 100 μm.

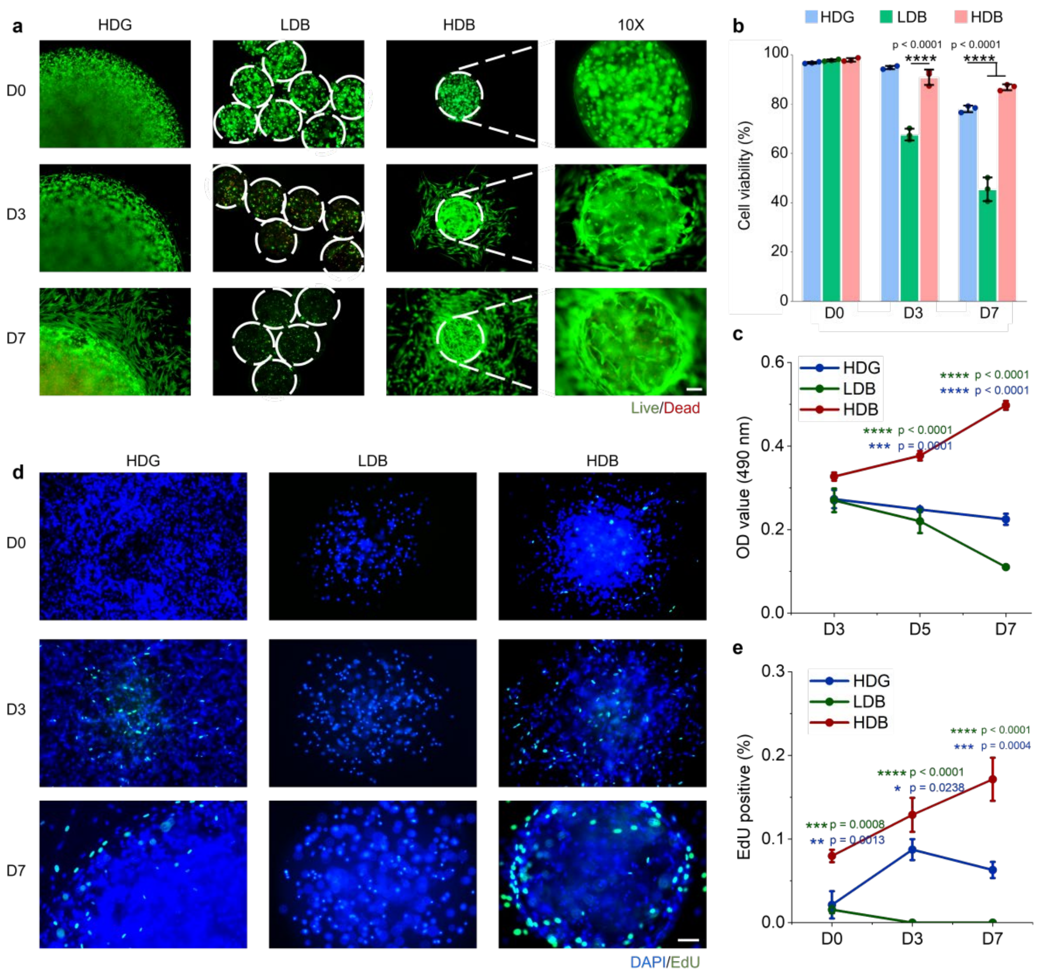

Figure S17. Characterization of MSC viability, migration and proliferation in the gelatin HDG, LDB and HDB groups. **a**, Representative images of MSCs with the live/dead staining reagents cultured in the gelatin HDG, LDB and HDB groups. **b,** MSC viability quantification at day 0 (D0), D3 and D7. n = 3. The green indicates the live cells, and the red means dead cells. **c**, OD values at 490 nm of proliferative MSCs cultured in the gelatin HDG, LDB and HDB groups at D3, D5 and D7. n = 3. **d,** EdU labeling of MSCs cultured in the gelatin HDG, LDB and HDB groups. **e,** EdU positive cells quantification at D0, D3 and D7. n = 3. Scale bars, 100 μm. All data are represented as mean ± SD. Significant difference is determined by one-way ANOVA followed by Tukey's test. Significant differences of parallel groups are compared to the HDG group. *p < 0.05; **p < 0.01; ***p < 0.001; ****p < 0.0001.

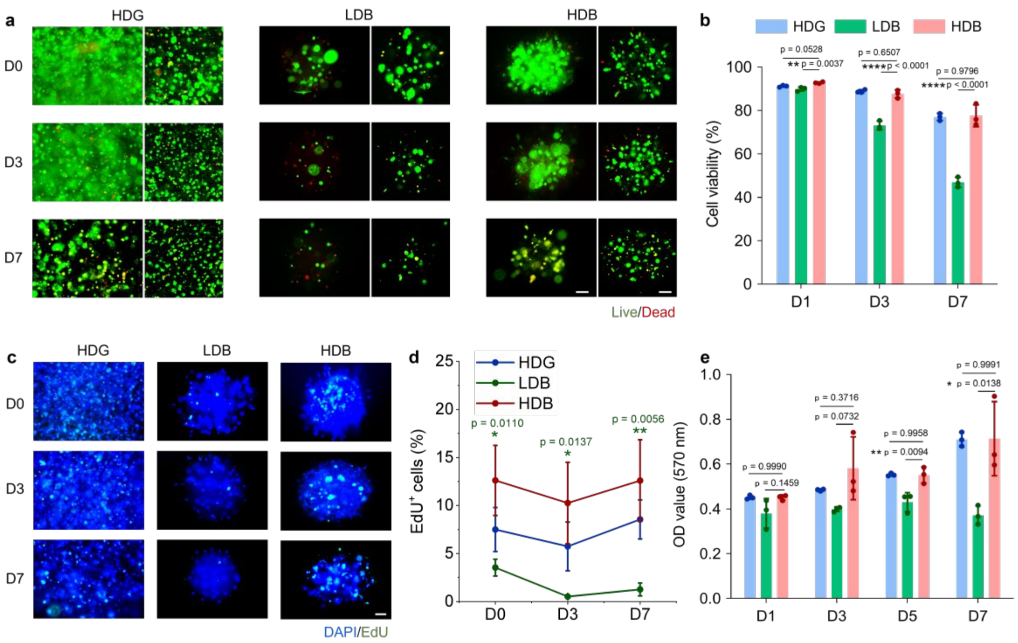

Figure S18. Characterization of MSC viability, migration and proliferation in the HAMA HDG, LDB and HDB groups. a, b, Representative live/dead fluorescence images (**a**) and viability rate quantification (**b**) of MSCs in the HAMA HDG, LDB and HDB groups at day 0 (D0), D3 and D7. **c-d,** Representative EdU staining images (**c**) and quantification (**d**) of MSCs in the HAMA HDG, LDB and HDB groups at D0, D3 and D7. **e**, Metabolic activity of MSCs in the HAMA HDG, LDB and HDB groups at D1, D3, D5 and D7, determined using the alamar blue assay. n = 3. The data are represented as mean ± SD. Significant difference is determined by one-way ANOVA, followed by Tukey's test. *p < 0.05; **p < 0.01; ****p < 0.0001. Scale bar, 100 μm in all panels.

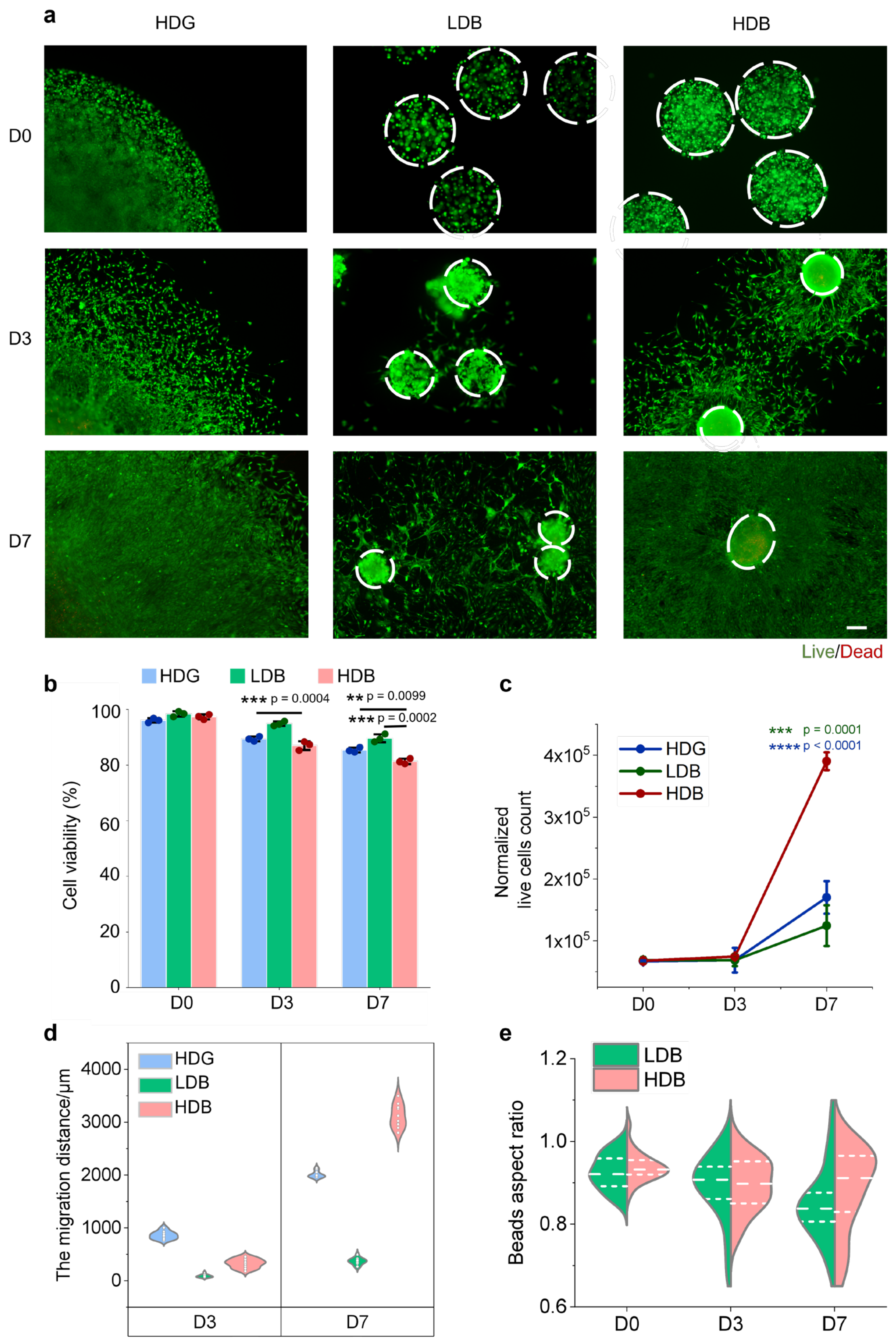

Figure S19. Characterization of NIH3T3 cells cultured in the Matrigel HDG, LDB and HDB groups. a, **b**, Representative live/dead staining images of NIH3T3 cells (**a**) and cell viability quantification (**b**) at day 0 (D0), D3, and D7. n = 3. Live cells were stained by Calcein-AM (green)

and dead cells were stained by propidium iodide (red). Scale bar, 200 μm. **c**, NIH3T3 live cell counting using hemocytometer at D0, D3, and D7. n = 3. **d**, Quantification of NIH3T3 cell migration distance at D3, and D7. n = 55 at each time point. **e**, Aspect ratio analysis of the Matrigel LDB and HDB beads at D0, D3, and D7. n = 55 at each time point. **b, c,** Data are represented as mean ± SD. Significant difference is determined by one-way ANOVA, followed by Tukey's test. Significant differences of all parallel groups are compared with the HDB group. *p < 0.05; ***p < 0.001; ****p < 0.0001.

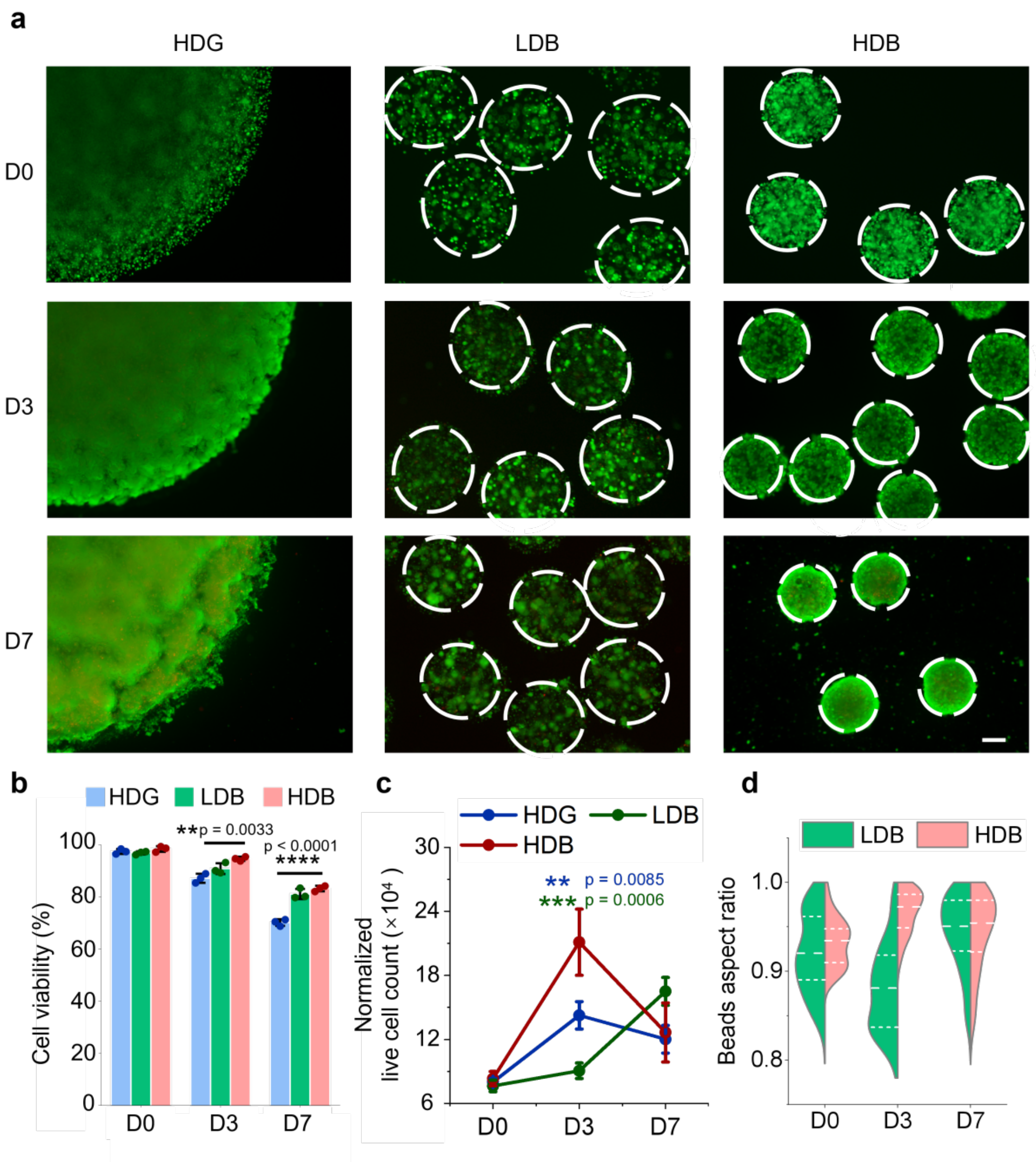

Figure S20. Characterization of SW620 cells cultured in the Matrigel HDG, LDB and HDB groups. **a**, **b**, Representative live/dead staining images of SW620 cells (**a**) and cell viability quantification (**b**) at day 0 (D0), D3, and D7. n = 3. Live cells were stained by Calcein-AM (green) and dead cells were stained by propidium iodide (red). Scale bar, 200 μm. **c**, NIH3T3 live cell counting using hemocytometer at D0, D3, and D7. n = 3. **d**, Aspect ratio analysis of the Matrigel LDB and HDB beads at D0, D3, and D7. n = 40 at each time point. **b, c,** Data are represented as mean ± SD. Significant difference is determined by one-way ANOVA followed by Tukey's test. Significant differences of all parallel groups are compared with the HDB group. $*p < 0.05$; $**p < 0.01$; $***p < 0.001$.

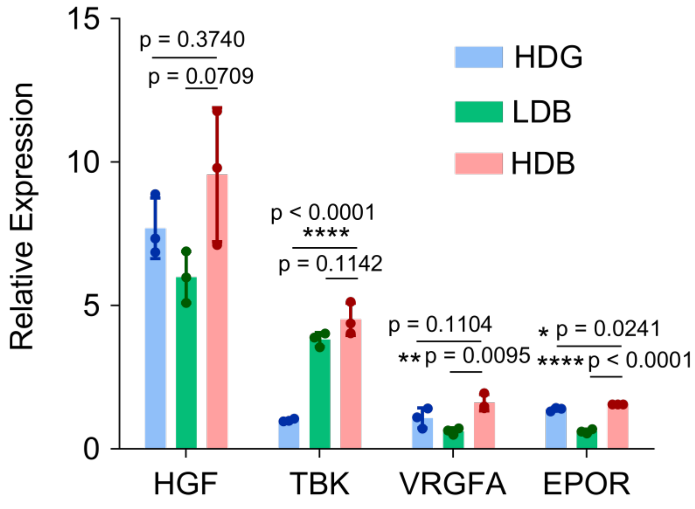

Figure S21. qPCR gene relative expression in the Matrigel HDG-MSCs, LDB-MSCs, HDB-MSCs groups, including HGF, TBK, VRGFA, and EPOR. n = 3 for each group. Hydrogel samples were from 3 different batches and used to perform the qPCR experiment. For each batch, three replicates were used and the average value was calculated to plot the data. The data are represented as mean ± SD. Significant difference is determined by one-way ANOVA, followed by Tukey's test. The significant differences of parallel groups are compared with the HDB group. *$p < 0.05$; ****$p < 0.0001$.

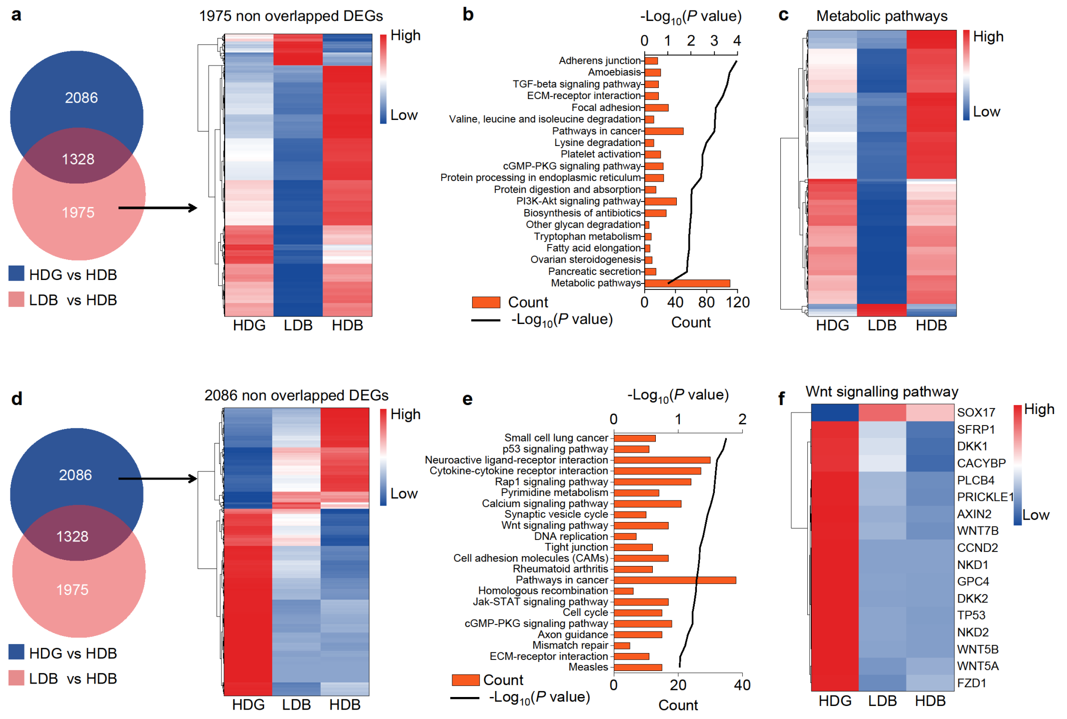

Figure S22. The RNA-seq analysis of non-overlapped genes of MSCs cultured in the Matrigel HDG group and the Matrigel LDB group, both compared to the Matrigel HDB group. **a**, Gene expression levels in non-overlapped region in the Matrigel LDB group versus the Matrigel HDB group. **b**, The KEGG pathway of 1975 non-overlapped differentially expressed genes in the Matrigel LDB group versus the Matrigel HDB group. Significant differential genes were determined using one-sided wald test in DESeq2 package. **c**, 110 Differentially expressed genes in the non-overlapped region involved in the metabolic pathway in the Matrigel LDB group versus the Matrigel HDB group. **d**, Gene expression levels in non-overlapped region in the Matrigel HDG group versus the Matrigel HDB group. **e**, The KEGG pathway of 2086 non-overlapped differentially expressed genes in the Matrigel HDG group versus the Matrigel HDB group. Significant differential genes were determined using one-sided wald test in DESeq2 package. **f**, Heatmap view of 17 Differentially expressed genes in the non-overlapped region involved in the Wnt signaling pathway in the Matrigel HDG group versus the Matrigel HDB group.

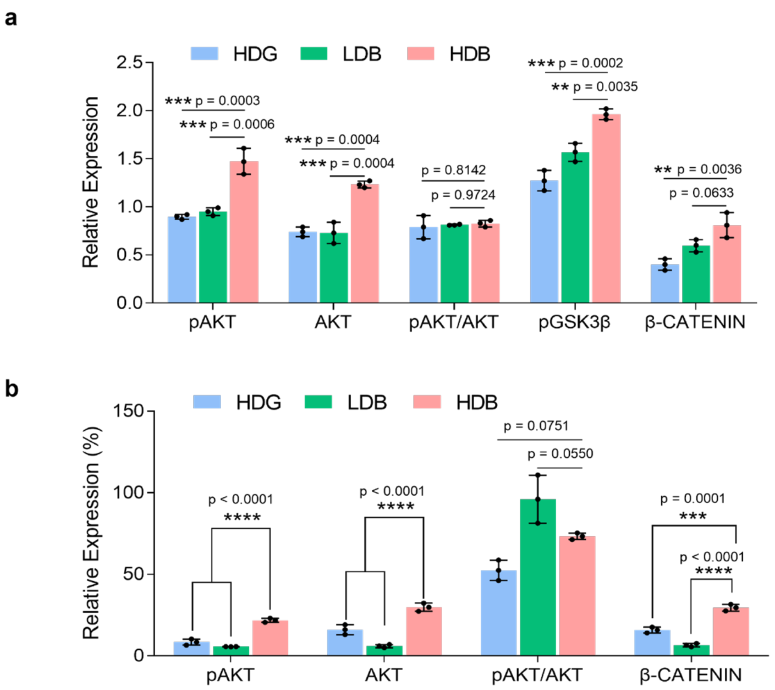

Figure S23. Quantification of activated proteins among the Matrigel HDG, LDB and HDB groups. **a**, Quantification of Western blot protein levels of MSCs. n = 3. **b**, Quantification of immunofluorescence for pAKT, AKT and β-CATENIN of MSCs. n = 3. Data are represented as mean ± SD. Significant difference is determined by one-way ANOVA followed by Tukey's test. Significant differences of all parallel groups are compared with the HDB group. *p < 0.05; **p < 0.01; ***p < 0.001;****p < 0.0001.

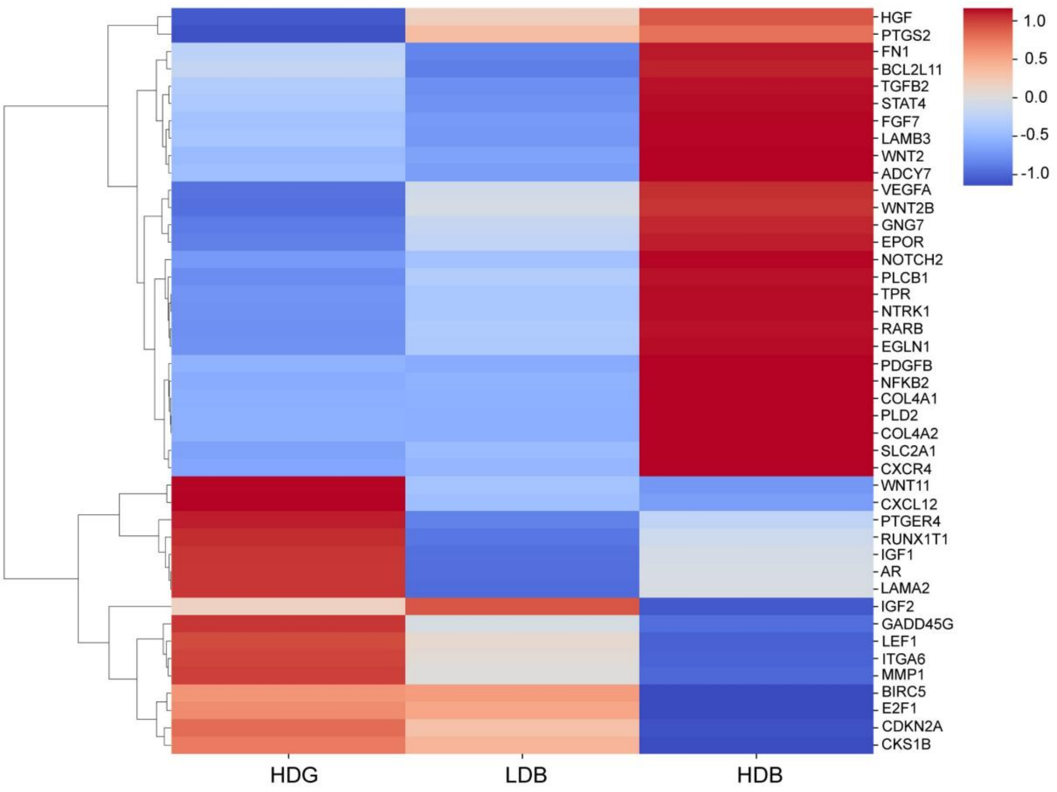

Figure S24. Heatmap of genes expression related to pathways in cancer in the Matrigel HDG, LDB and HDB-MSCs.

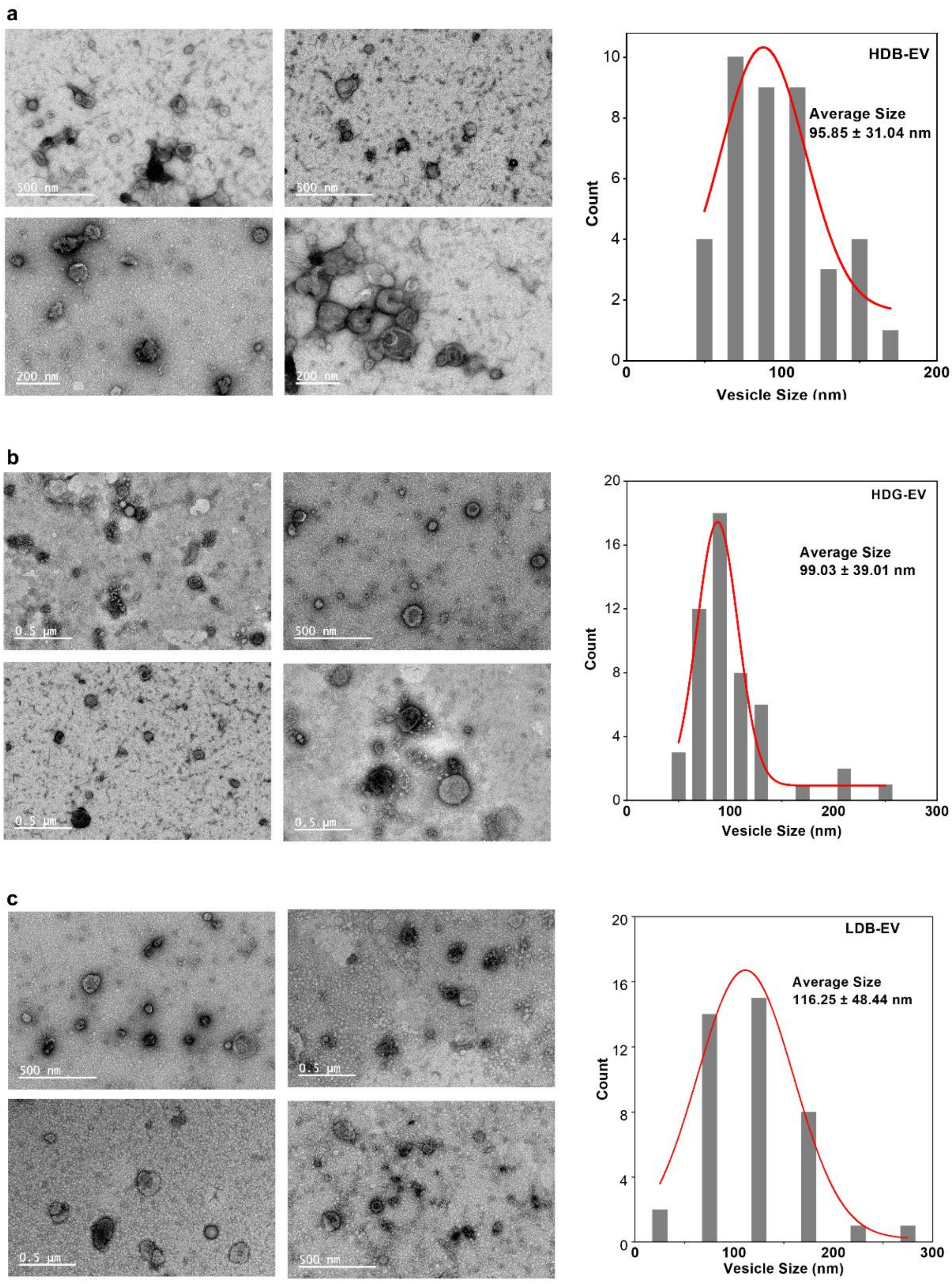

Figure S25. TEM images and the corresponding particle size distribution curves for HDB-EVs (a), HDG-EVs (b) and LDB-EVs (c).

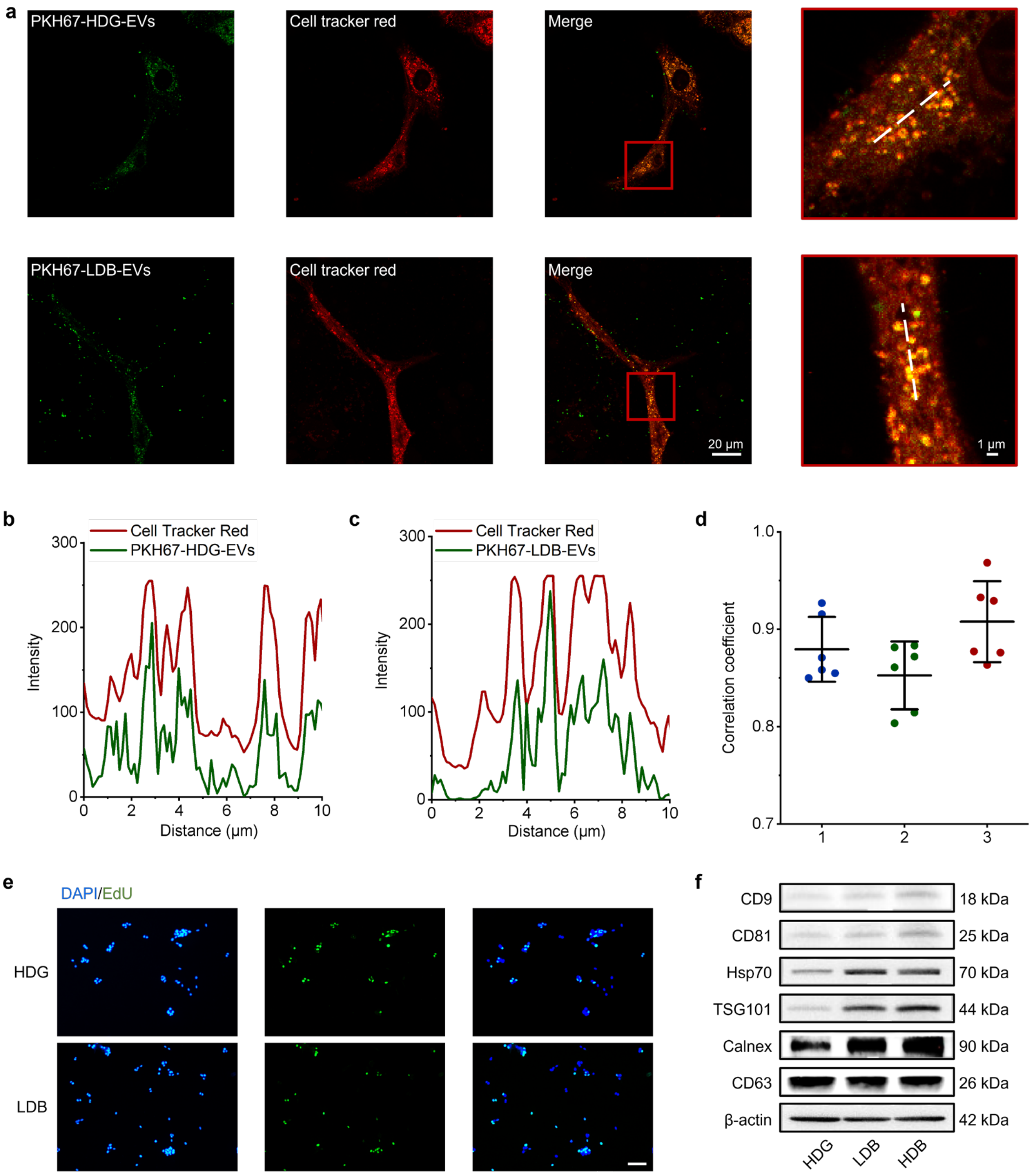

Figure S26. Characteristics of LDB-EVs and HDB-EVs. a, Colocalization between HDG-EVs, LDB-EVs and C2C12 cells. PKH67-labeled HDG-EVs and LDB-EVs were incubated with C2C12 for 48 hours. Cells were stained with Dil cell membrane trackers. **b-c**, Intensity profiles of signals from the two fluorescent channels along the dashed lines, respectively. C2C12 cells were cocultured with EV derived from HDG-MSCs (**b**) and LDB-MSCs (**c**). **d**, Quantification of mean Pearson's correlation coefficients recorded using ImageJ. n = 3. 6 regions of interest (ROIs) were analyzed. Data are represented as mean ± SD. **e**, Representative images of EdU staining of C2C12 cells treated by 10 μg/ml HDG-EVs and LDB-EVs. Scale bars, 100 μm. **f**, Western blotting of EV marker proteins (CD9, CD81, Hsp70, TSG101, Calnex, CD63) in MSCs cultured in Matrigel HDG, LDB

and HDB groups. Samples were derived from the same experiment and the gels were processed in parallel.

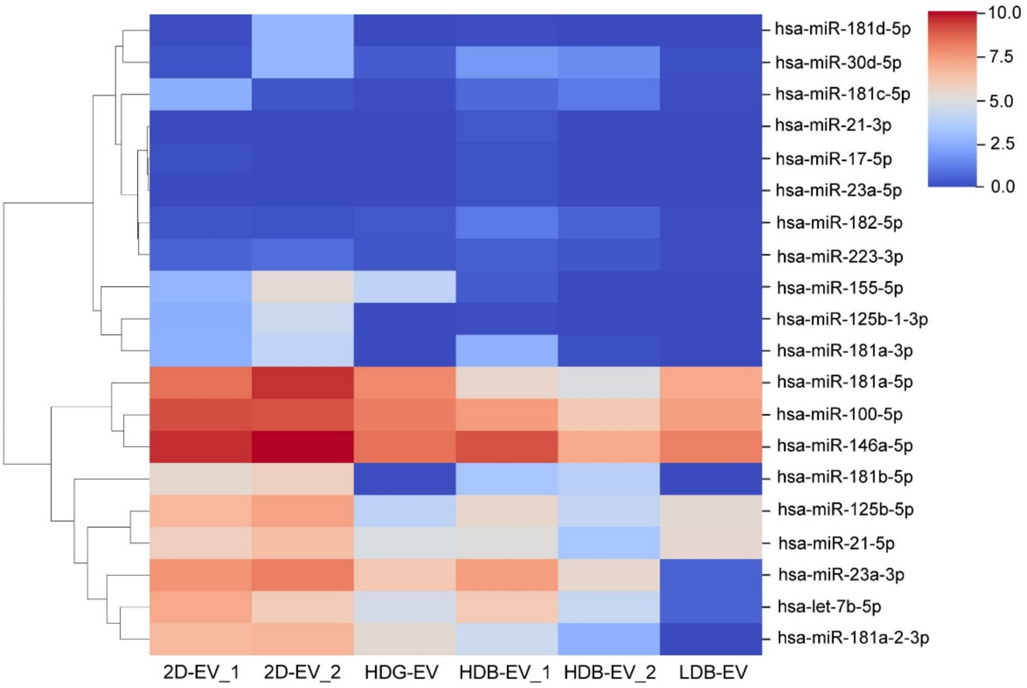

Figure S27. Heatmap of genes expression related to anti-inflammatory activities in EVs derived from 2D-culture, and the Matrigel HDG, Matrigel LDB, Matrigel HDB groups.

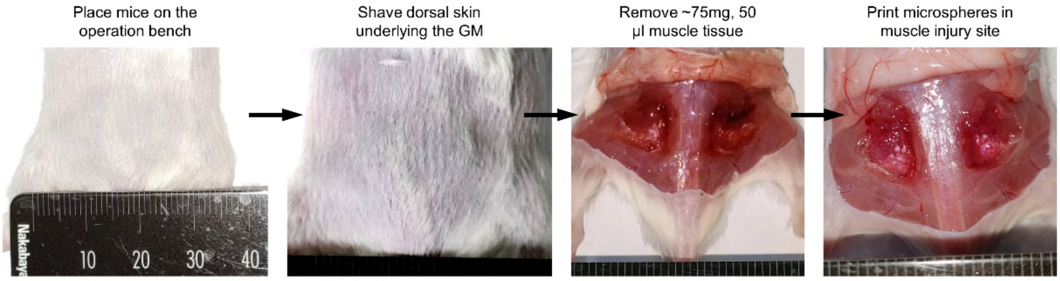

Figure S28. Surgical procedure of GM VML modeling.

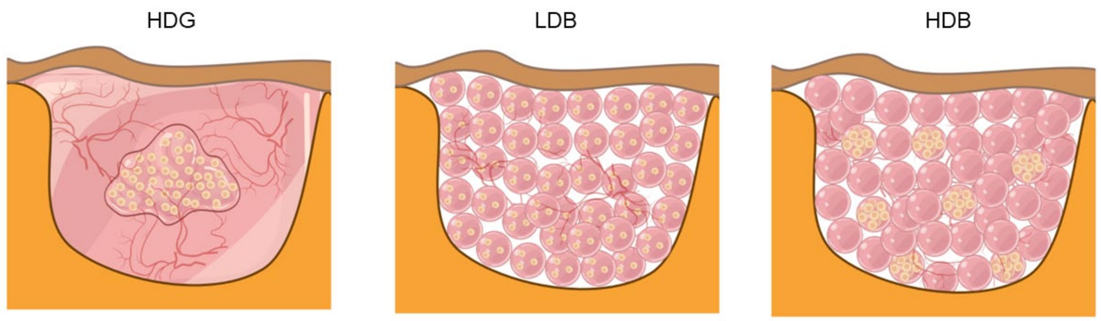

Figure S29. HDG-MSCs, LDB-MSCs, HDB-MSCs as printed or casted to fill the defect volume.

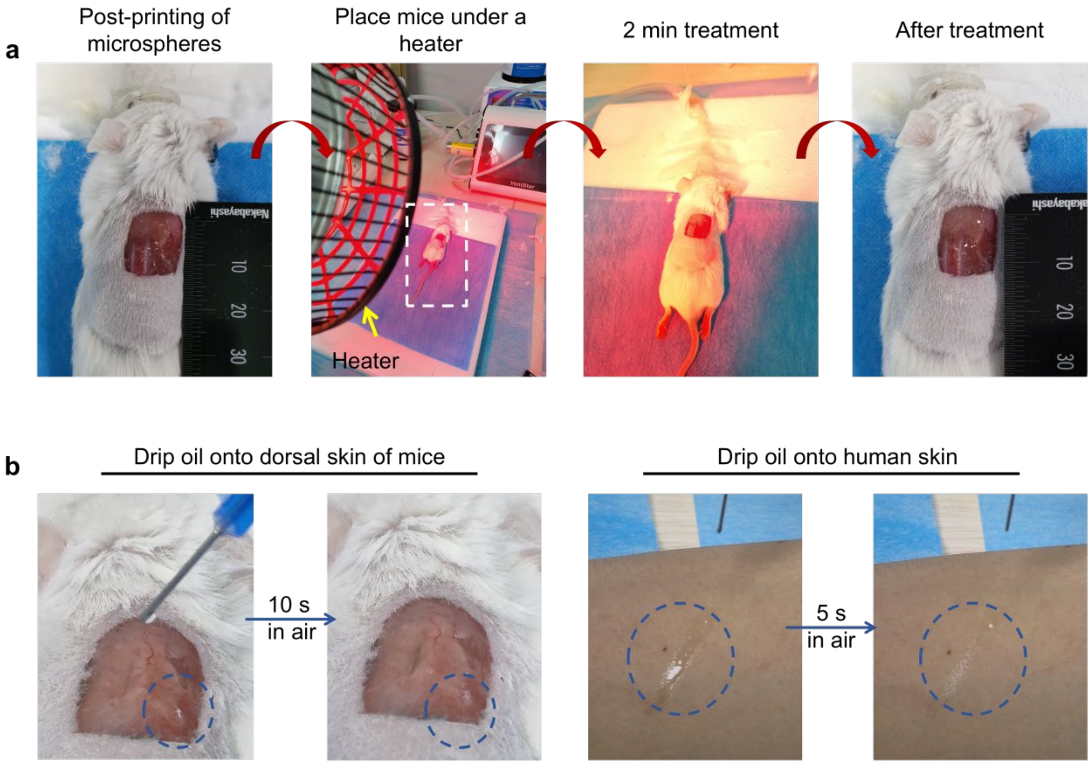

Figure S30. Oil evaporation test. a, A mouse was placed under a heater for 2 min. **b**, Drip oil onto the dorsal skin of a shaved mouse and human skin.

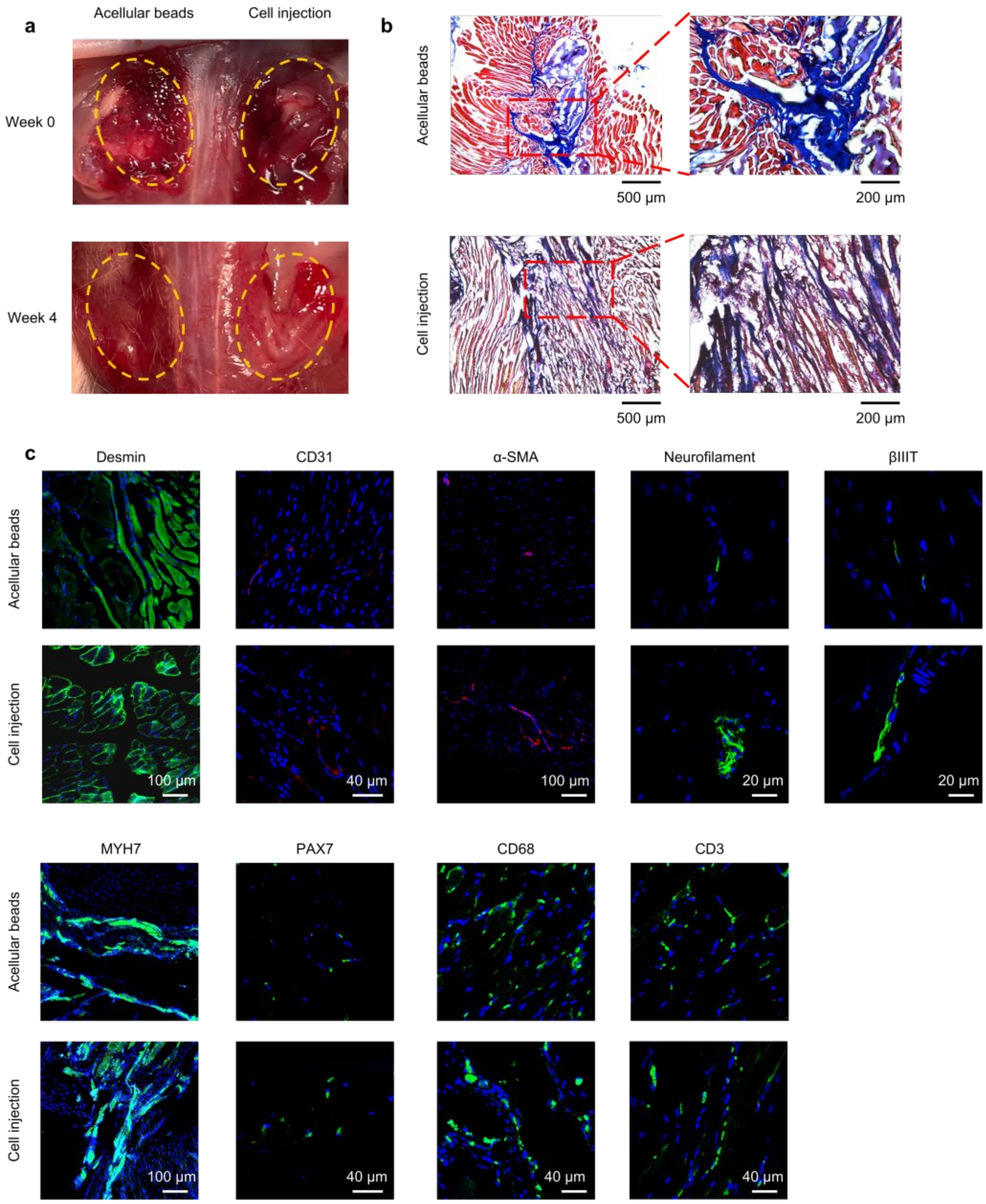

Figure S31. Muscle regeneration treated with acellular Matrigel beads and MSCs suspension injection. The acellular beads were densely printed as in the practice of HDB plus acellular beads, and MSCs suspension were injected in the whole VML region. **a**, The photograph of acellular beads and cell injection post-printing and regeneration after 4 weeks. **b**, The MTS staining of muscle tissues harvested at week 4 treated using acellular beads and MSCs injection. **c**, Representative images of immunostaining for desmin, CD31, α-SMA, neurofilament, βIIIT, MYH7, PAX7, CD68 and CD3. The injected MSCs were identical to the MSCs-HDB implantation in cell counts, with 9 × 10^4 cells per wound.

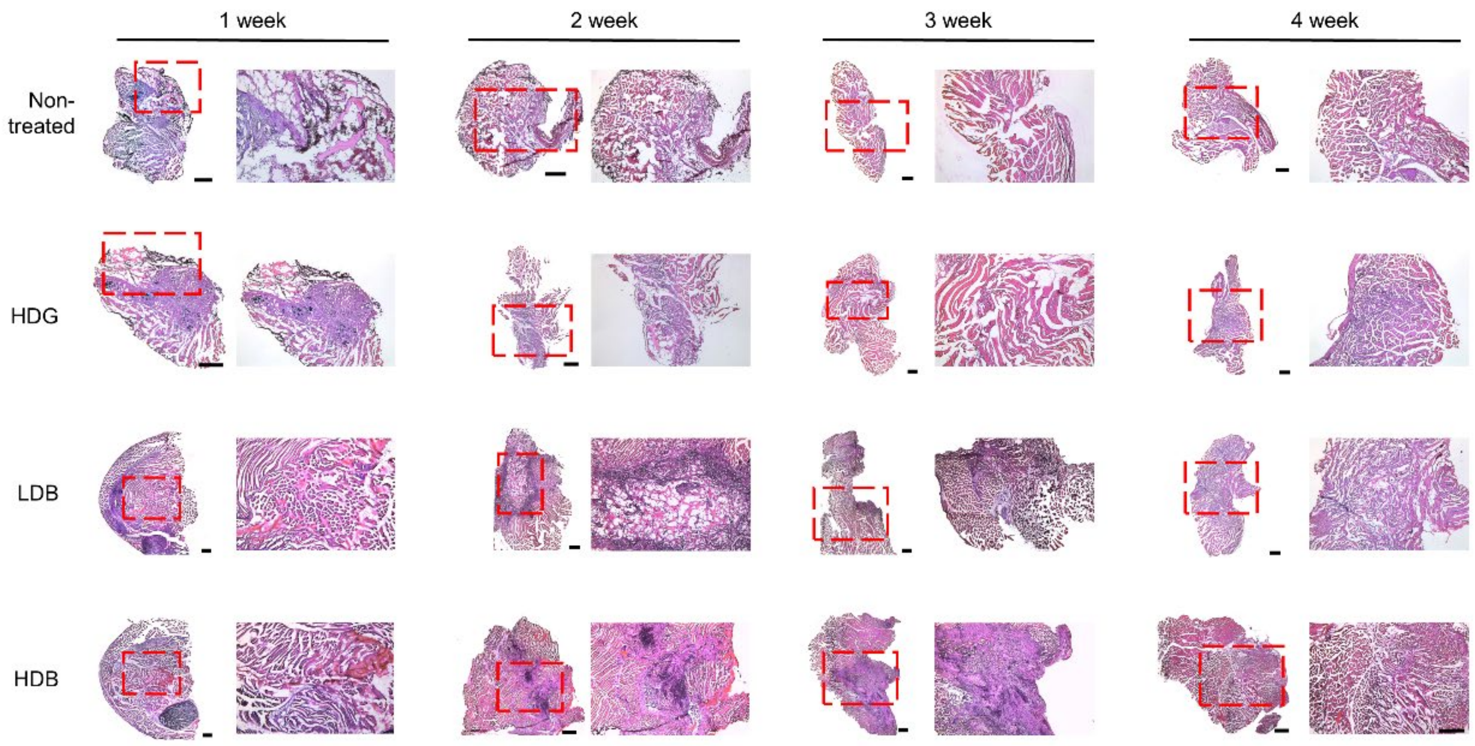

Figure S32. The full H&E and magnification images of regenerated muscle tissues at 1 week, 2 weeks, 3 weeks and 4 weeks, in mice VML model with no treatment (self-healing), casted with MSCs-laden Matrigel HDG, and printed with MSCs-laden LDB and HDB beads, respectively. The HDB-MSCs were printed in sparse pattern, interspaced with acellular Matrigel beads. Scale bars, 500 µm.

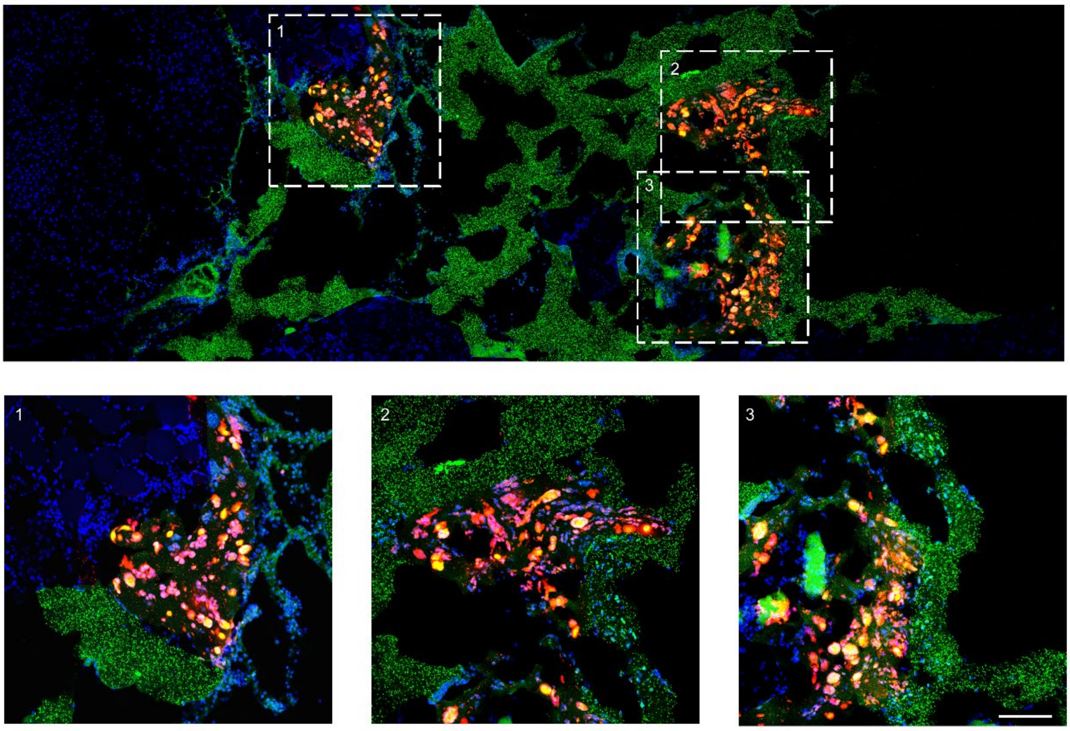

Figure S33. Matrigel MSCs-HDB complex and acellular beads were distributed in the muscle defect at D7 after implantation. Matrigel MSCs-HDB was surrounded by acellular beads. MSCs were labeled using the red Dil cell membrane tracker (Meilunbio). Acellular beads and acellular bulk gel were loaded with monodispersed green fluorescent polystyrene microspheres (Aladdin, 1 μm). Scale bar, 100 μm.

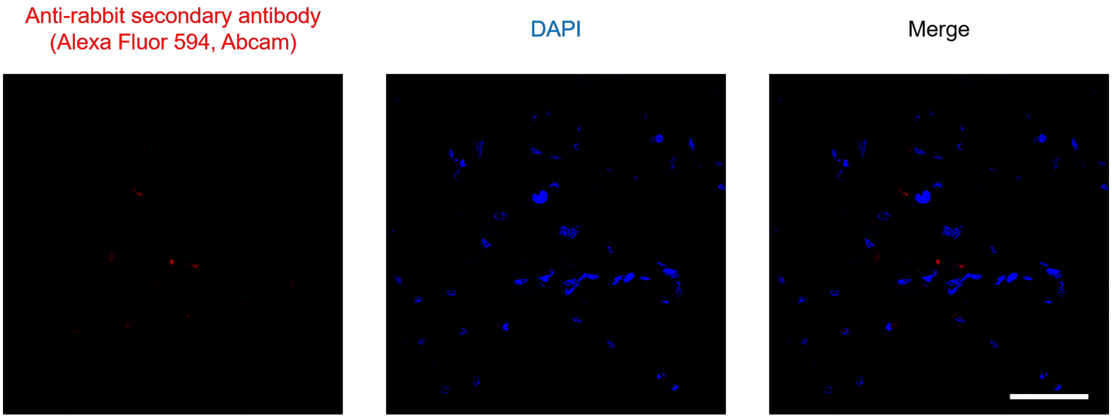

Figure S34. Representative images of negative control stained using anti-rabbit secondary antibody (Alexa Fluor 594) without HLA primary in Matrigel HDB-MSCs after 4-week transplantation. Scale bar, 50 μm.

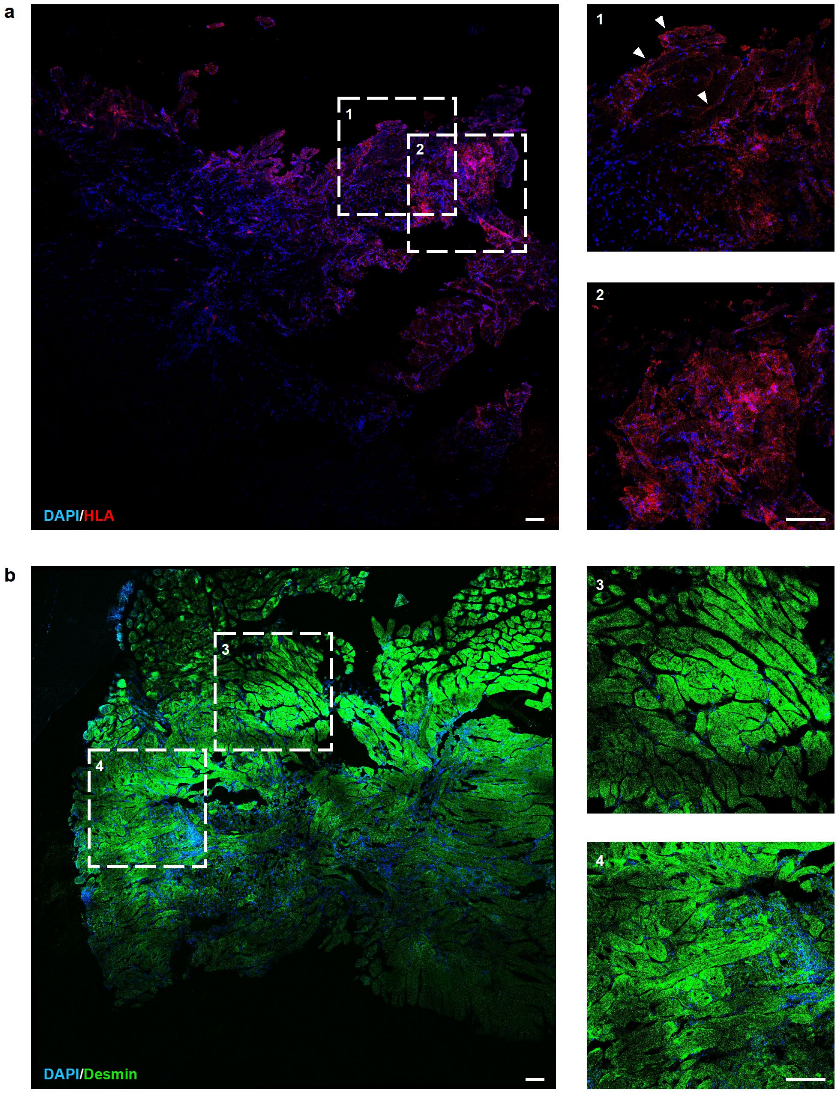

Figure S35. Immunofluorescence for Desmin and HLA of retrieved muscle tissues at week 4, treated by Matrigel HDB-MSCs. a, Immunofluorescence for HLA. Windows 1 and 2 show the zoom-in view of HLA intensive regions. The white arrow shows the HLA^+ signals with myofiber-like features. **b**, Immunofluorescence for Desmin. Windows 3 and 4 show the zoom-in view of developed and developing muscle tissues. Scale bars, 100 µm.

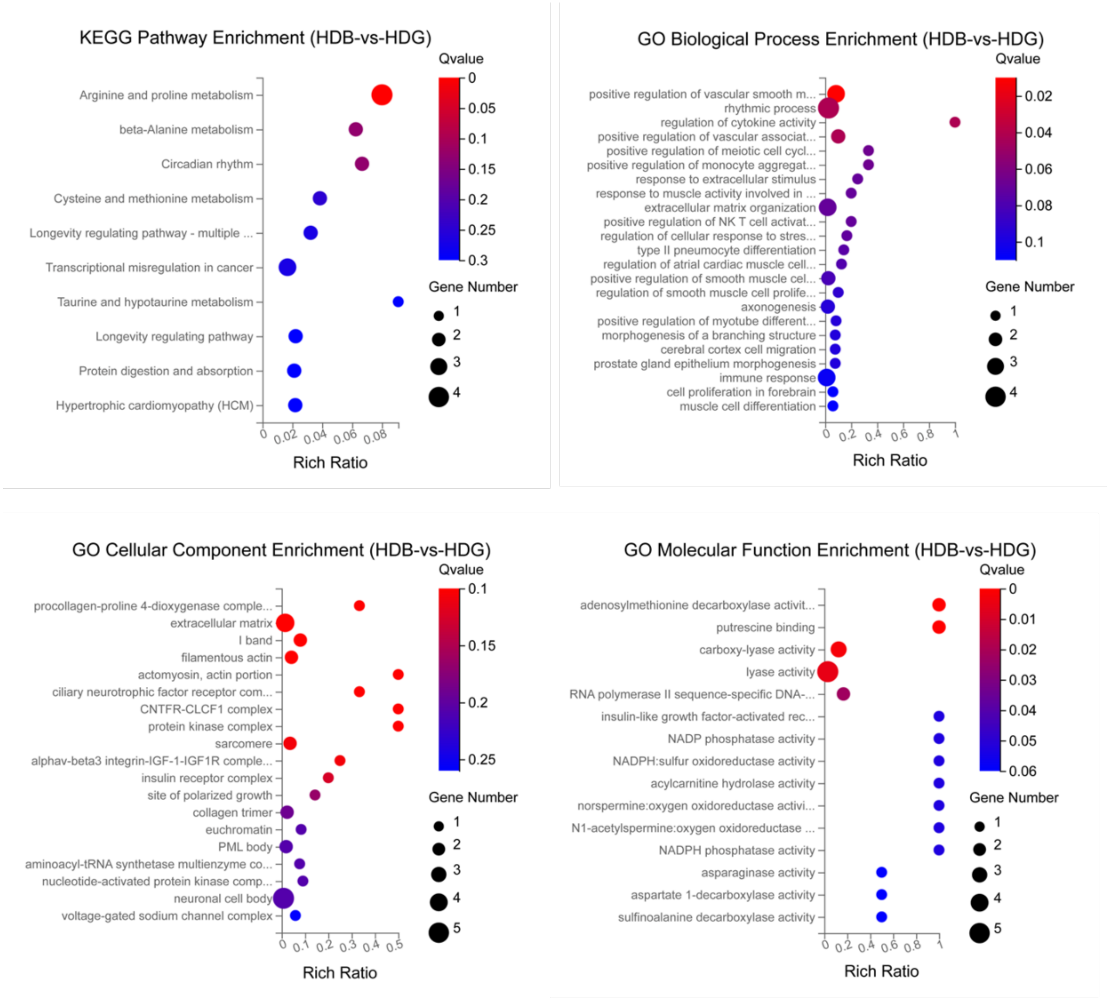

Figure S36. KEGG and GO analysis of RNA-seq data of regenerated muscle tissues. Injured muscle tissues were repaired by transplanting MSCs in HDB and HDG.

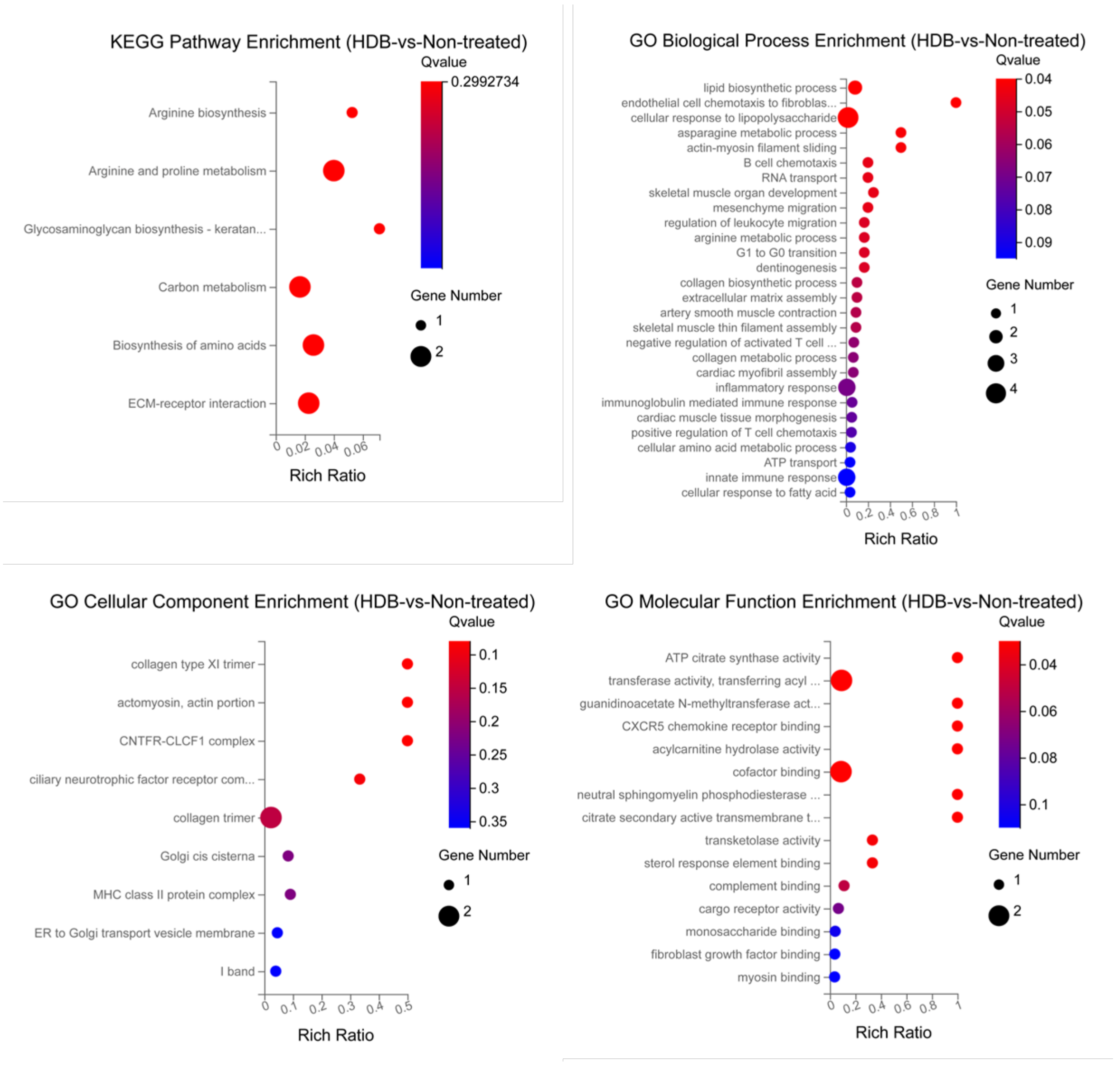

Figure S37. KEGG and GO analysis of RNA-seq data of regenerated muscle tissues. Injured muscle tissues were repaired by transplanting MSCs in HDB or self-repaired (the non-treat group).

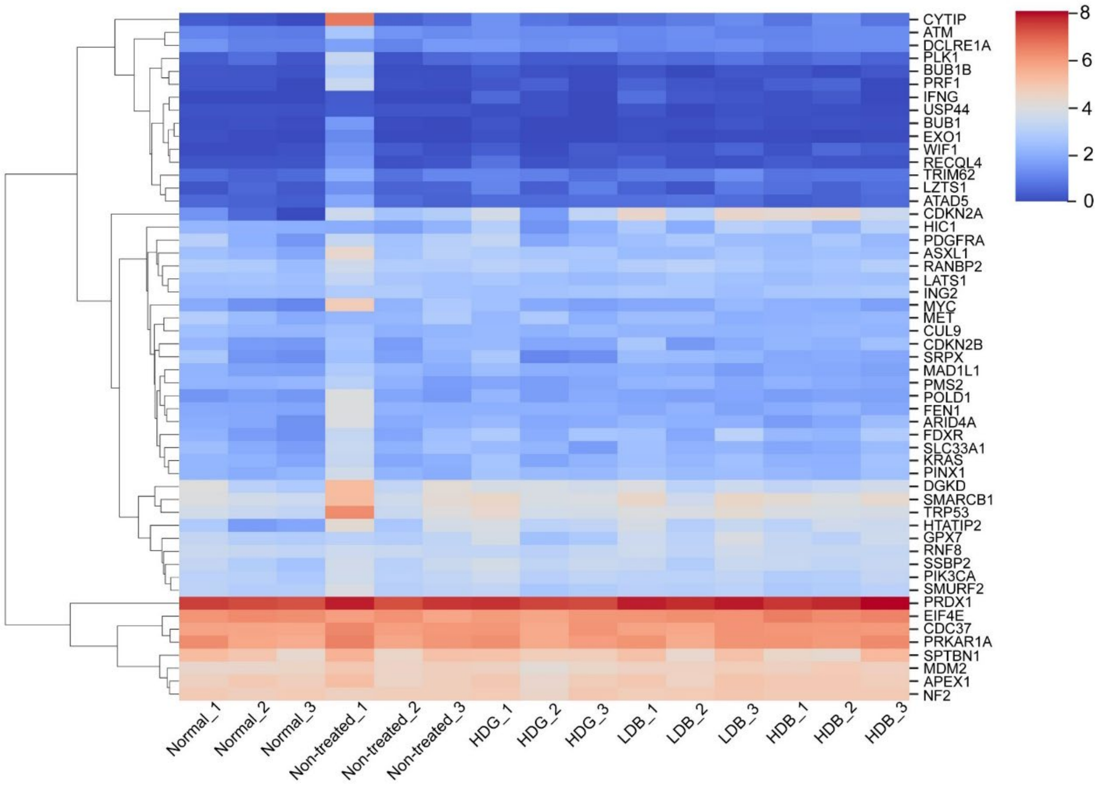

Figure S38. Heatmap of genes expression related to malignant transformation of RNA-seq of regenerated muscle tissues. Injured muscle tissues were repaired by transplanting MSCs in HDG, LDB, HDB or self-repaired, i.e. the non-treated group.

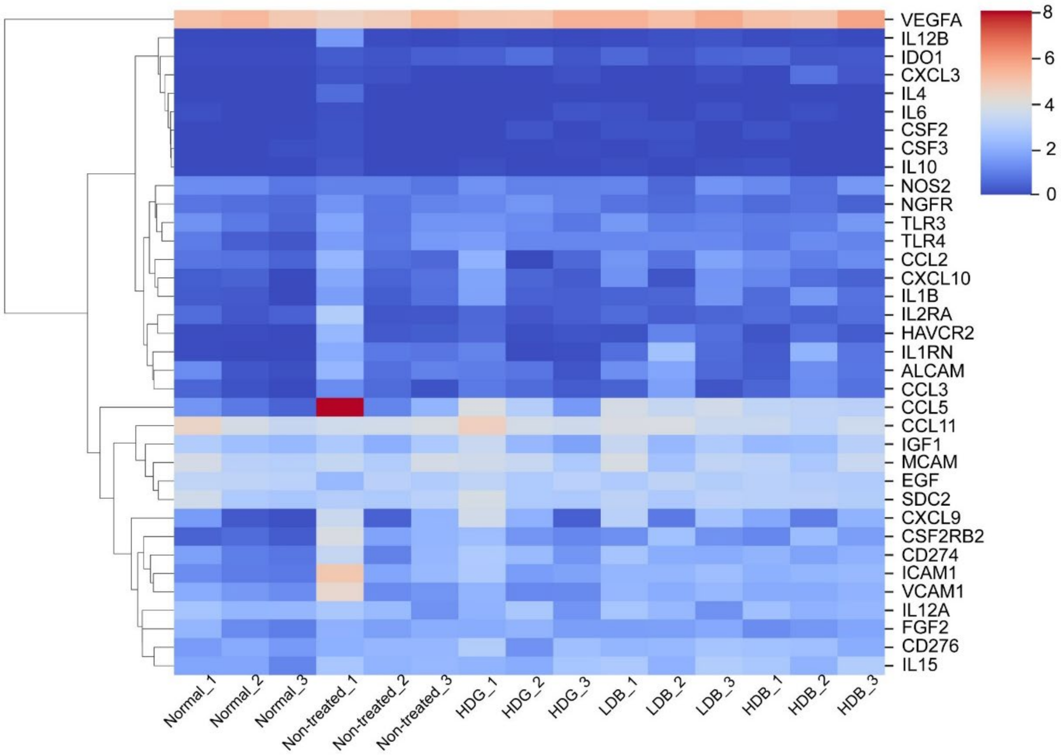

Figure S39. Heatmap of genes expression related to MSCs secretome of RNA-seq of regenerated muscle tissues by transplanting Matrigel HDG, Matrigel LDB, Matrigel HDB, and self-healing (non-treated) and normal groups.

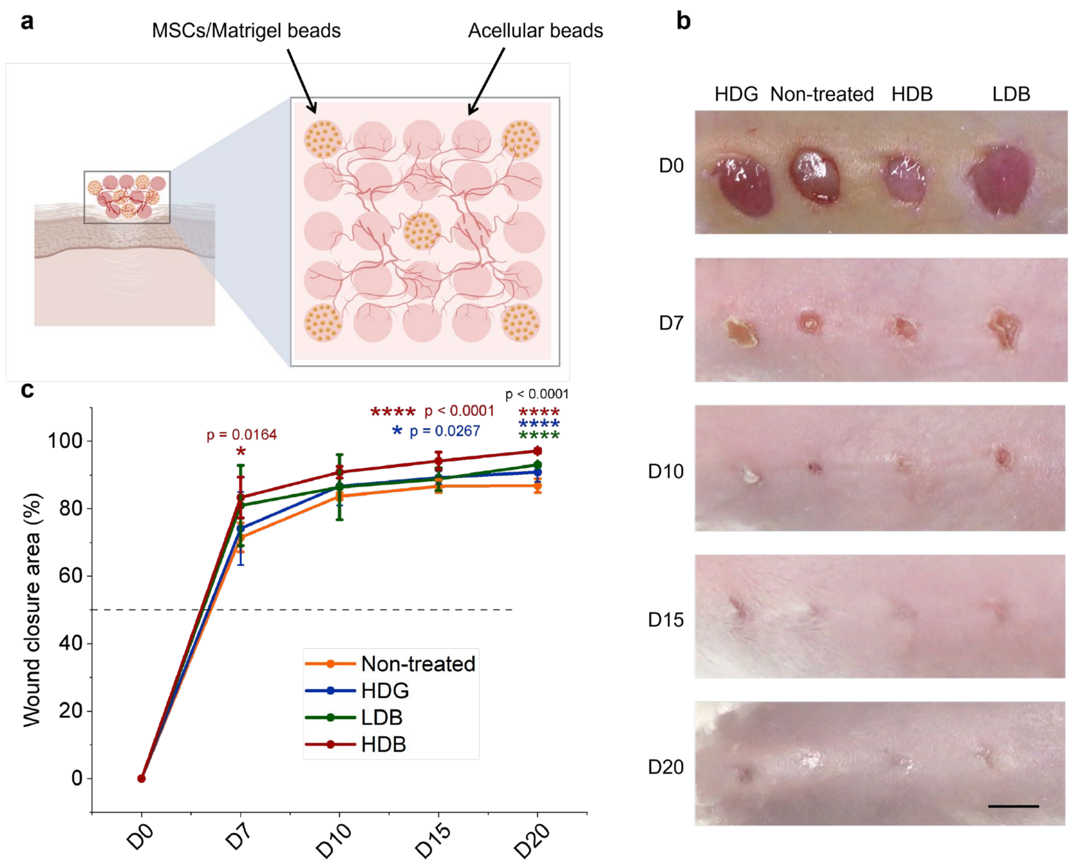

Figure S40. The skin wound healing process by self-healing (Non-treated), or treated with Matrigel HDG, LDB and HDB-MSCs. a, The schematic illustration of the wound post-printing of Matrigel HDB-MSCs beads. The wound volume was sparsely printed with HDB beads and interspaced with acellular Matrigel beads. **b-c**, The photographs of wound closure area (**b**) and quantification (**c**) at day 0 (D0), D7, D10, D15 and D20, respectively. n = 6 at each time point. Scale bar, 5 mm. The data are represented as mean ± SD. Significant difference is determined by one-way ANOVA, followed by Tukey's test. Significant differences of all parallel groups are compared with the non-treated group. *p < 0.05; **p < 0.01; ***p < 0.001.

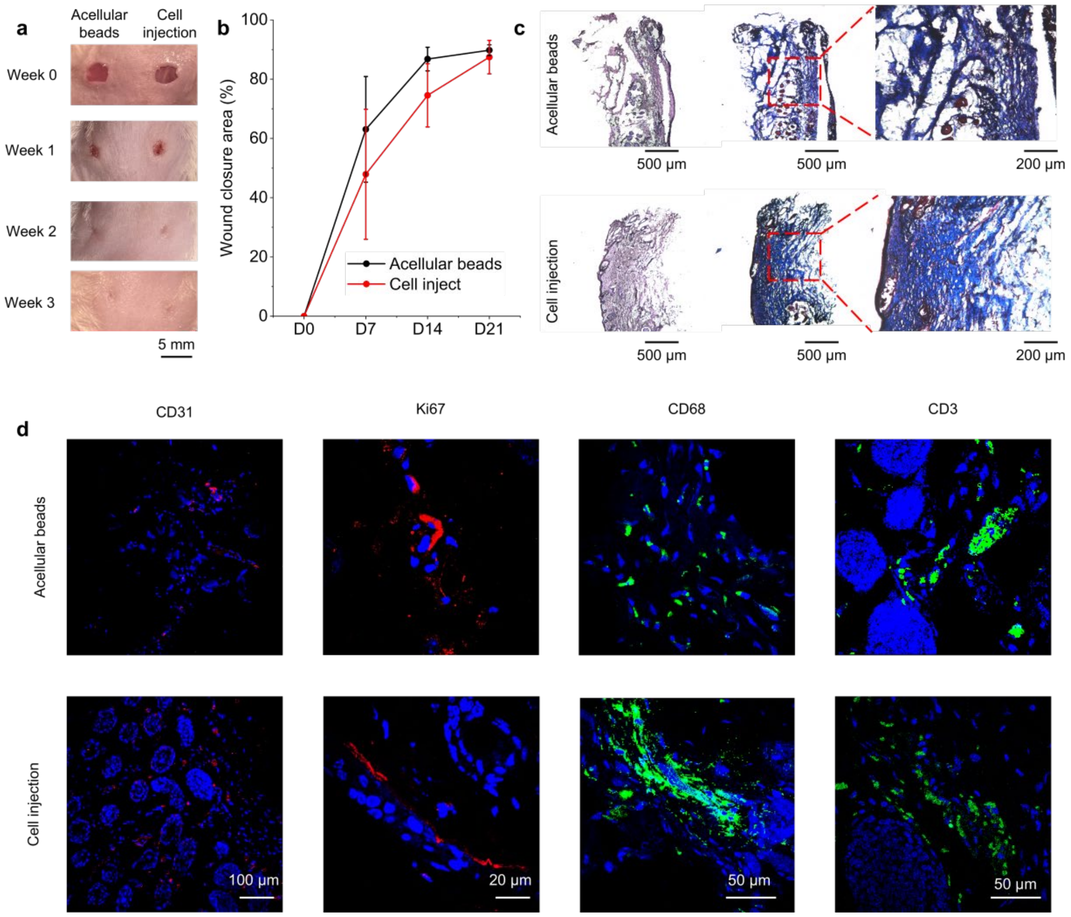

Figure S41. Skin wound regeneration treated with acellular Matrigel beads and MSCs suspension injection. The acellular beads were densely printed as in the practice of HDB plus acellular beads and MSCs suspension were injected in the skin wound region. **a-b**, The photographs of wound closure area (**a**) and quantification (**b**) at week 0, week 1, week 2, week 3. (n = 3 per group). Data are represented as mean ± SD. **c,** The HE and MTS staining of skin tissues harvested at week 3 treated using acellular beads and MSCs injection. **d,** Representative images of immunostaining for CD31, Ki67, CD68, CD3. The injected MSCs were identical to the MSCs-HDB implantation in cell counts, 2.1×10^4 cells per wound.

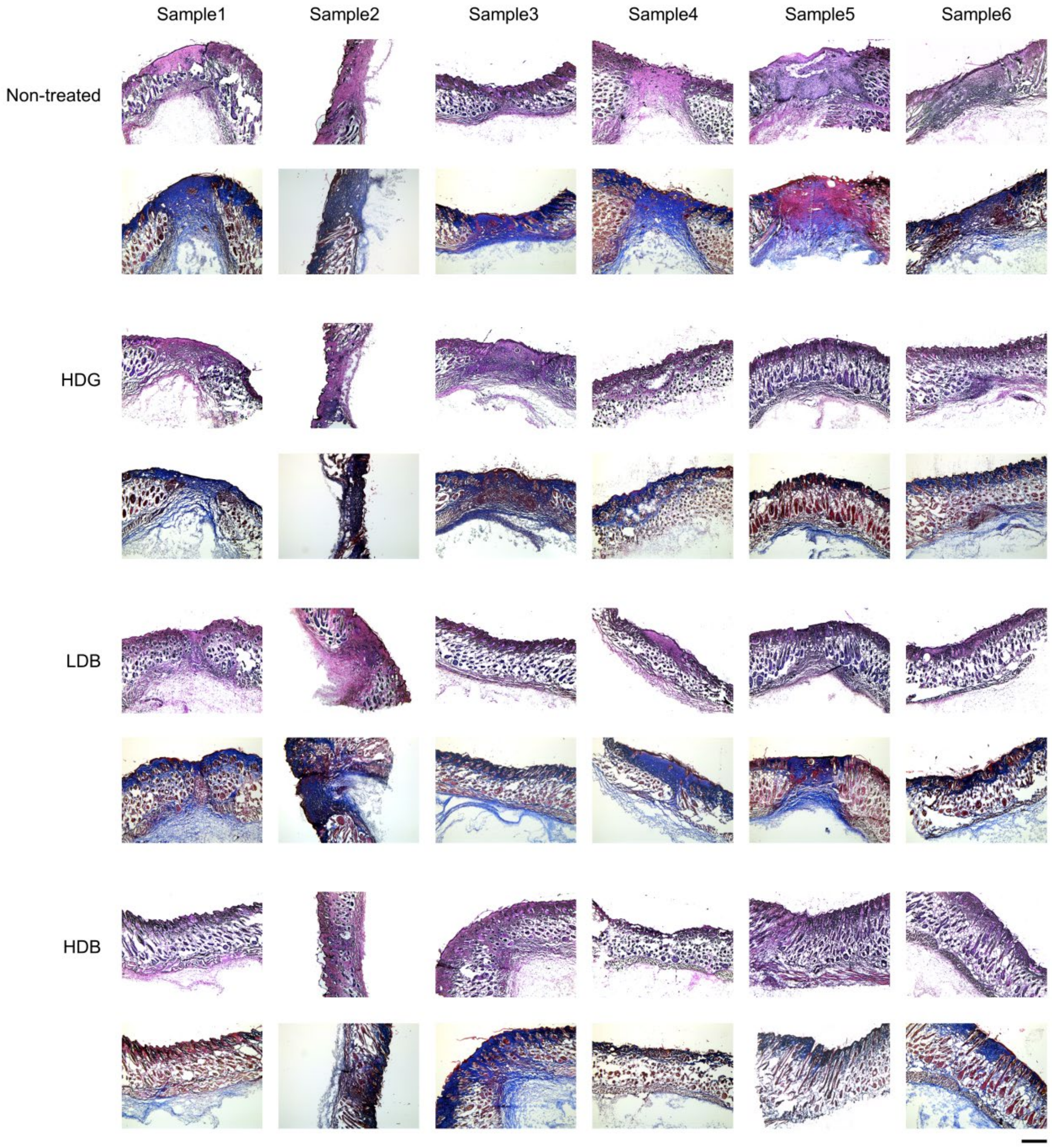

Figure S42. The H&E and MTS staining of 6 skin tissue samples after self-healing (Non-treated), or treated with Matrigel LDB-MSCs and HDB-MSCs at 3 weeks post-printing (or post-casting of Matrigel HDG-MSCs). Scale bar, 500 μm.

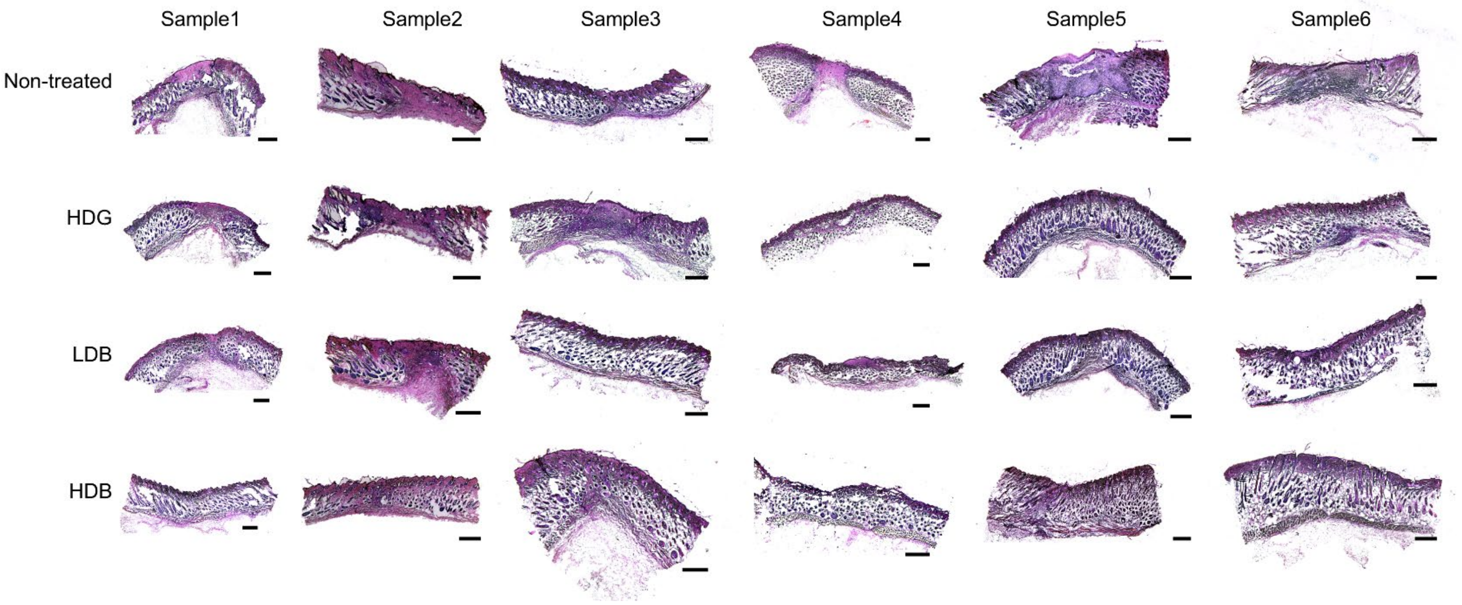

Figure S43. The full H&E image of 6 skin tissue samples after self-healing (Non-treated), or treated with Matrigel LDB-MSCs and HDB-MSCs at 3 weeks post-printing (or post-casting of Matrigel HDG-MSCs). Scale bars, 500 μm.

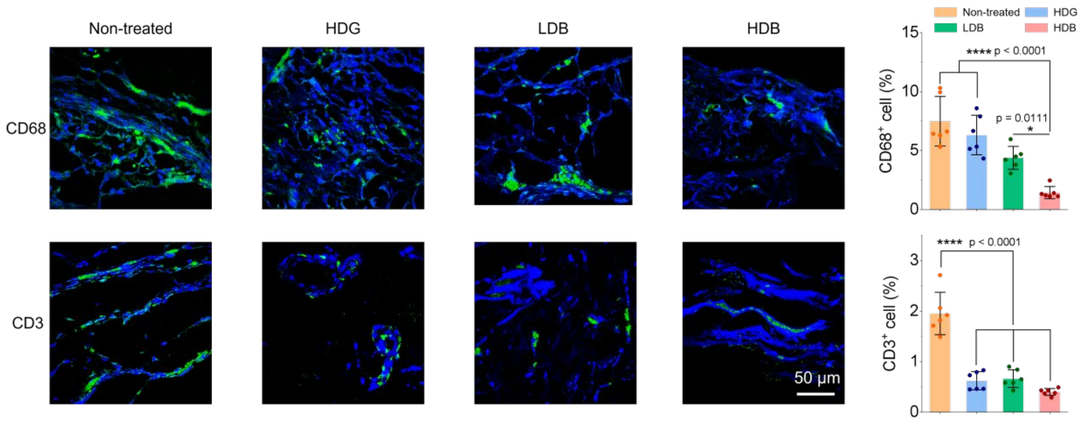

Figure S44. Immunofluorescence for CD68 and CD3 to determine the population of macrophages and T-cells and their quantification by fluorescence area in skin tissues. n = 6 per group at each time point. The data are represented as mean ± SD. Significant difference is determined by one-way ANOVA, followed by Tukey's test. *$p < 0.05$; ****$p < 0.0001$.

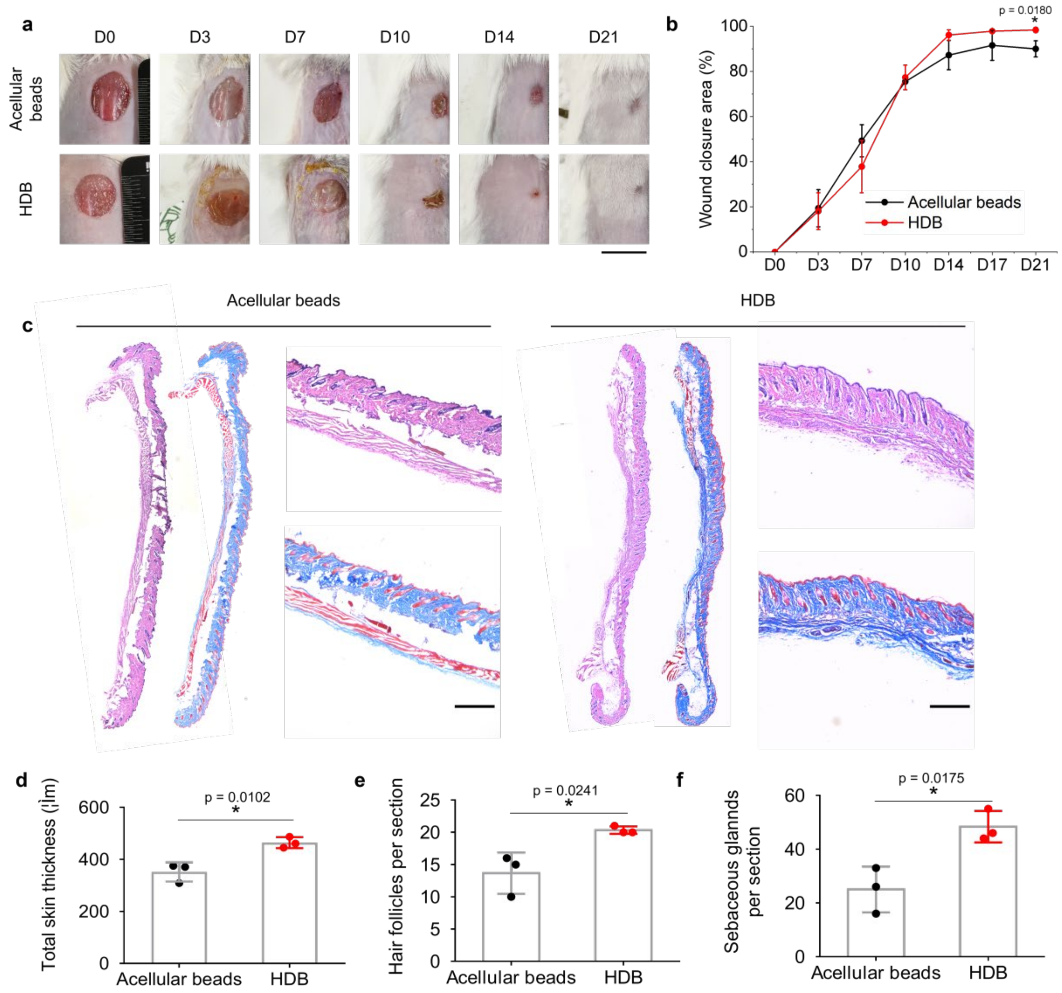

Figure S45. Matrigel HDB-MSCs augment skin wound (10 mm × 10 mm) healing and hair follicle regeneration. a-b, The photographs of wound closure area (**a**) and quantification (**b**) at day 0 (D0), D3, D7, D10, D14, and D21, respectively. n = 3 at each time point. Scale bar, 1 cm. **c**, Representative images of H&E staining and MTS of regenerated skin tissues at 4 weeks post-printing. Scale bar, 500 μm. n = 3 for each group. **d-f**, Quantification of total skin thickness (**d**), hair follicle (**e**), and sebaceous gland counts (**f**) per section in the regenerated area. n = 3. *p < 0.05. n = 3 for each group. The data are represented as mean ± SD. Significant difference is determined by unpaired two-failed student t-test. *P < 0.05.

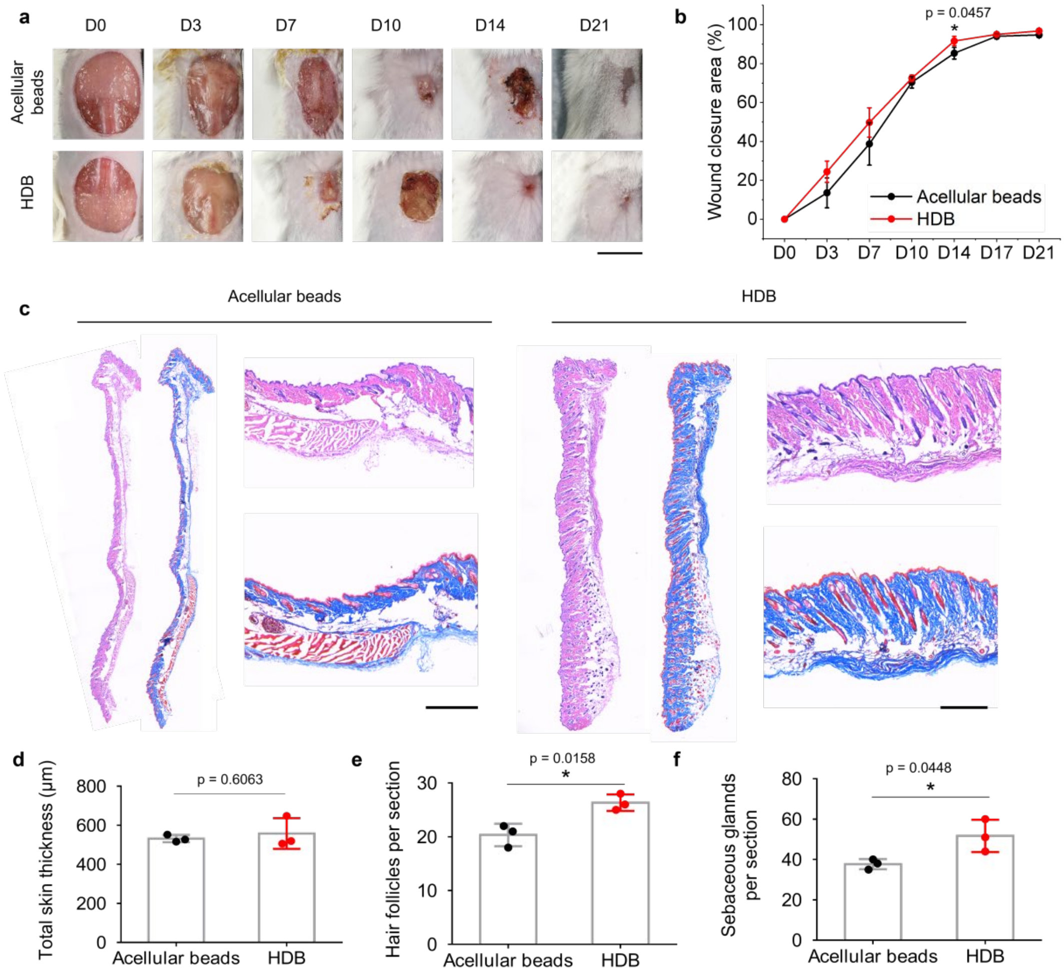

Figure S46. Matrigel HDB-MSCs augment LARGE skin wound (15 mm × 15 mm) healing and hair follicle regeneration. a-b, The photographs of wound closure area (**a**) and quantification (**b**) at D0, D3, D7, D10, D14 and D21, respectively. n = 3 at each time point. Scale bar, 1 cm. **c**, Representative images of H&E staining and MTS of regenerated skin tissues at 4 weeks post-printing. n = 3 for each group. Scale bars, 500 μm. **d-f**, Quantification of total skin thickness (**d**), hair follicle (**e**), and sebaceous gland counts (**f**) per section in the regenerated area. n = 3. n = 3 for each group. The data are represented as mean ± SD. Significant difference is determined by unpaired two-failed student t-test. *P < 0.05.

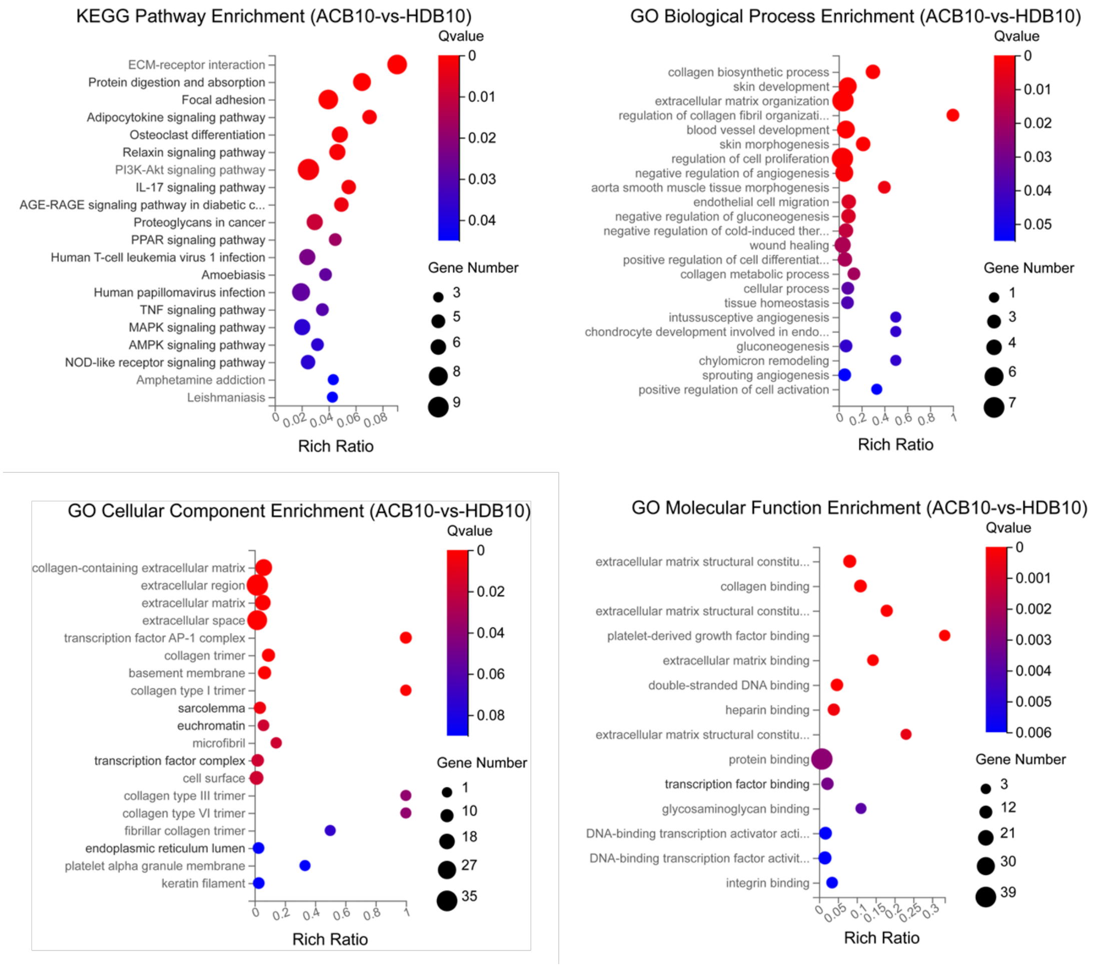

Figure S47. KEGG and GO analysis of RNA-seq data of regenerated skin tissues. 10-mm round full-thickness excisional wounds were regenerated by transplanting acellular beads (ACB) and MSCs-HDB.

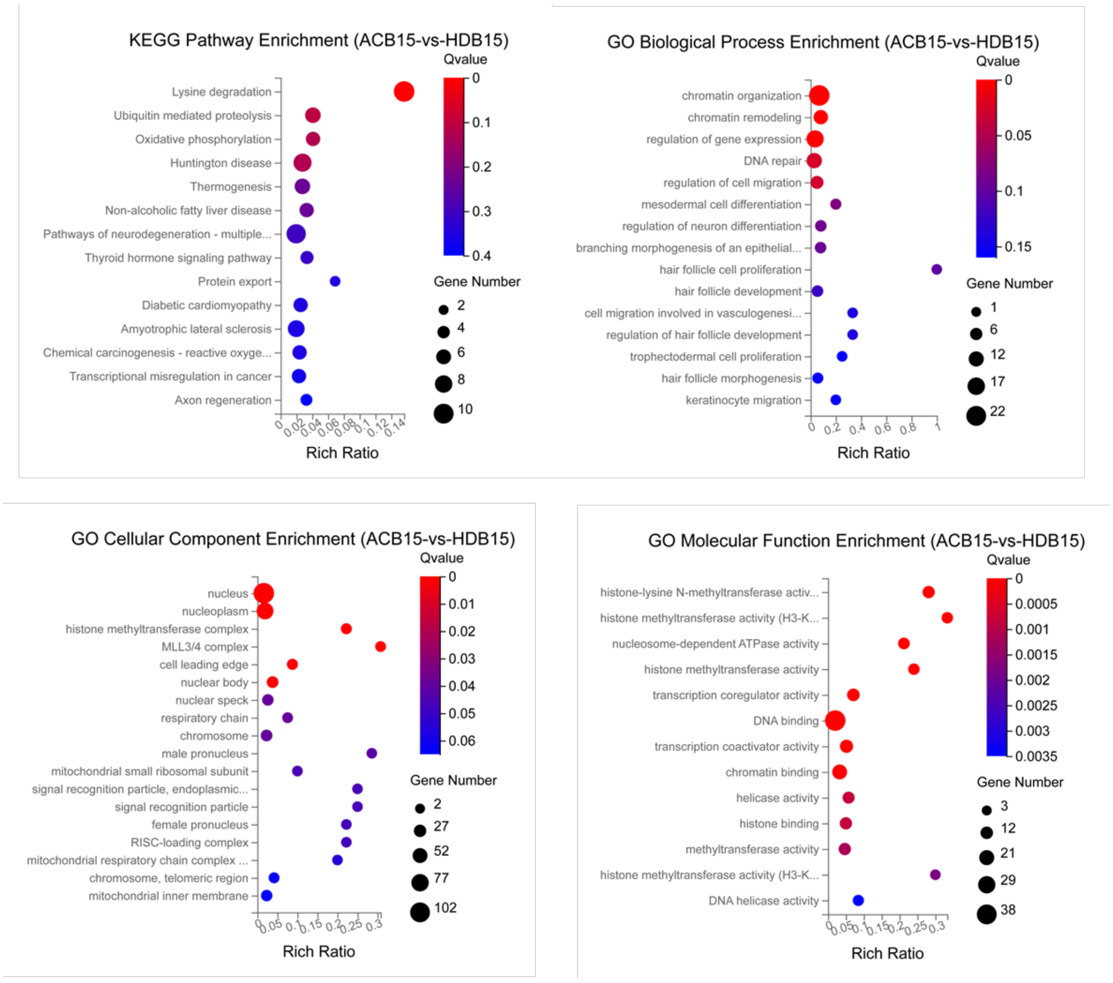

Figure R48. KEGG and GO analysis of RNA-seq data of regenerated skin tissues. 15-mm rounded full thickness excisional wounds were regenerated by transplanting acellular beads (ACB) and MSC-HDB.

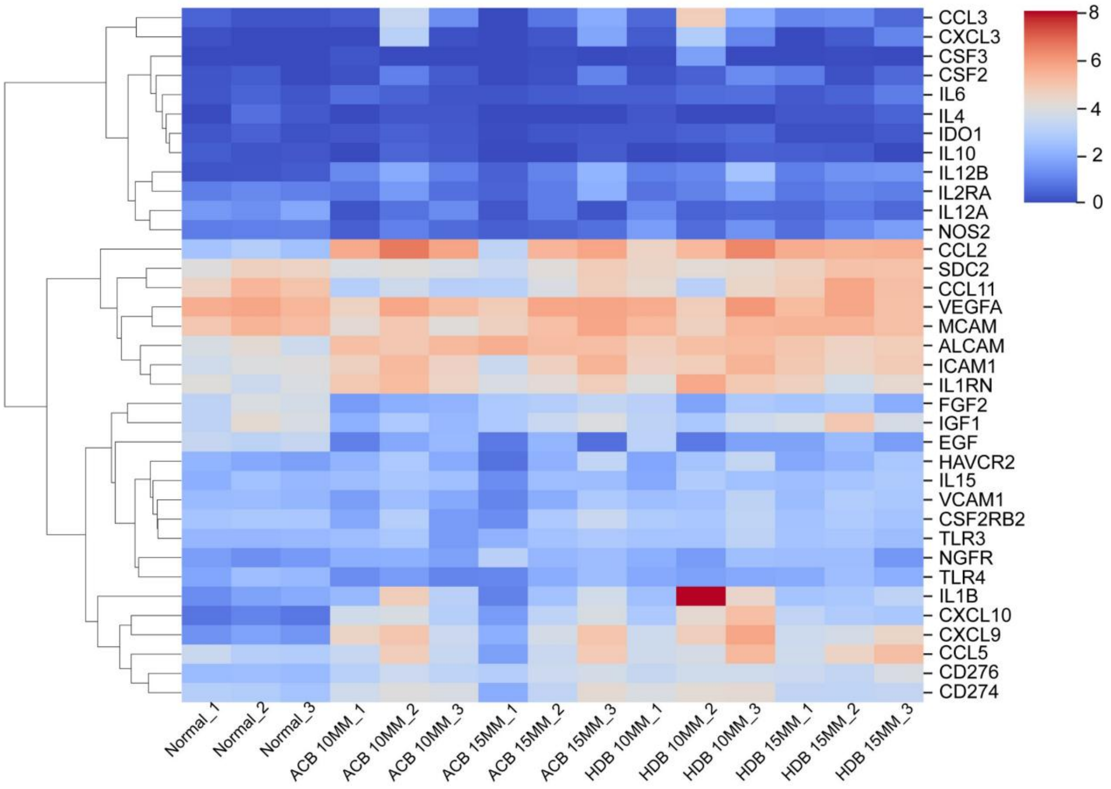

Figure S49. Heatmap of genes expression related to MSCs secretome in regenerated skin tissue regenerated by Matrigel HDB, acellular beads (ACB) and normal groups.

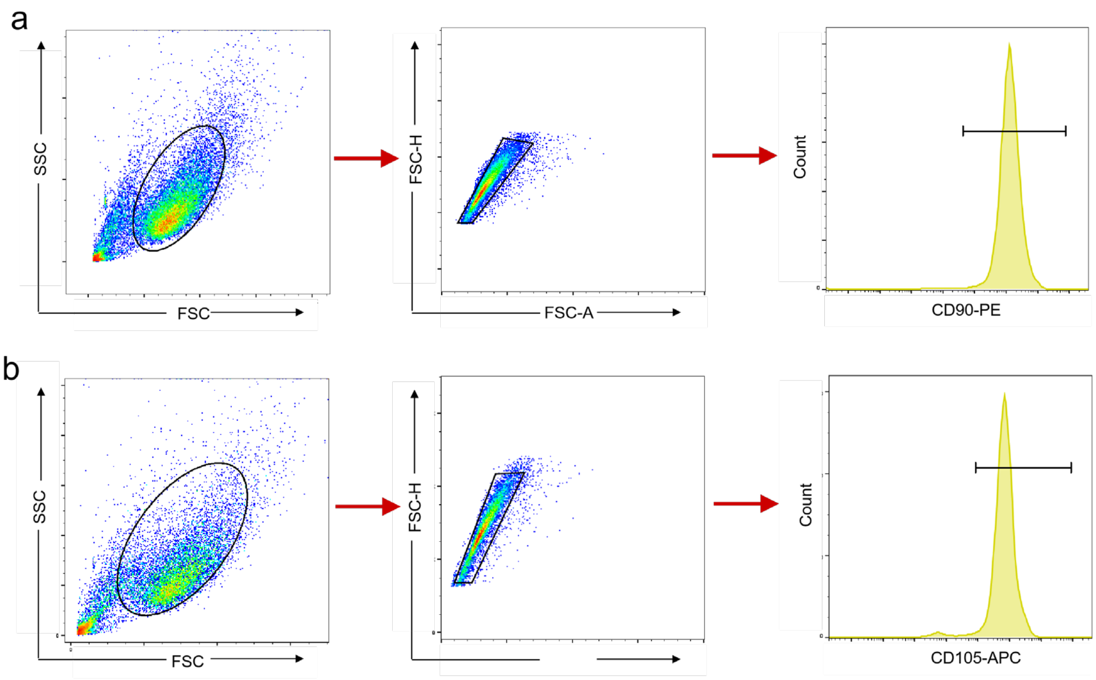

Figure S50. Gating strategies of CD90 (a) and CD105 (b) used in Fig. 3j.

Table S1. Number of cells in microbeads and in bulk gel.

Hydrogel formation	HDG	LDB	HDB
Cell density	1×10^7 cells/mL	2×10^6 cells/mL	1×10^7 cells/mL
Cells / bead volume	1×10^4 cells/μL	300 cells/bead	1500 cells/bead

Note. The number of cells in microbeads for dosage studies were given in the figure legends.

Table S2. Number of cells delivered at the wound sites

Tissue types	Volume and size	Total cell number	HDG group	LDB group	HDB group
VML GM tissues	60% volume (~ 75mg, 45 µl)	9 × 10^4 cells	9 µL HDGs, 36 µl acellular gel	300 LDBs	60 HDBs, 240 acellular beads
Skin wounds	5 mm × 5 mm	2.1 × 10^4 cells	2.1 µL HDGs, 8.4 µL acellular gel	70 LDBs	14 HDBs, 56 acellular beads
	10 mm × 10 mm	8.4 × 10^4 cells	8.4 µL HDGs, 33.6 acellular gel	280 LDBs	56 HDBs, 224 acellular beads
	15 mm × 15 mm	1.8 × 10^5 cells	18 µL HDGs, 72 µl acellular gel	600 LDBs	120 HDBs, 480 acellular beads

Supplementary Video 1. Bead-jet printing toward a high throughput 2d manner and 3d assembly.

Supplementary Video 2. Z-scan of granular gelatin microgels.

Supplementary Video 3. Z-scan of granular Matrigel microgels.

Supplementary Video 4. Z-scan of granular GelMA microgels.

Supplementary Video 5. Z-scan of granular HAMA microgels.

Supplementary Video 6. Bead-jet printing of Matrigel beads onto irregular mouse skin wound
