# Supplementary material for: Bead-jet printing enabled sparse mesenchymal stem cell patterning augments skeletal muscle and hair follicle regeneration
Source: Nat Commun. 2022 Dec 3;13:7463. doi: 10.1038/s41467-022-35183-8 (PMC9718784; doi:10.1038/s41467-022-35183-8)
Supplement: Supplementary file 3 — Description of additional Supplementary File [file 41467_2022_35183_MOESM3_ESM.pdf]

Descriptions of Additional Supplementary Files

Supplementary Video 1. Bead-jet printing toward a high throughput 2d manner and 3d assembly.

Supplementary Video 2. Z-scan of granular gelatin microgels.

Supplementary Video 3. Z-scan of granular Matrigel microgels.

Supplementary Video 4. Z-scan of granular GelMA microgels.

Supplementary Video 5. Z-scan of granular HAMA microgels.

Supplementary Video 6. Bead-jet printing of Matrigel beads onto irregular mouse skin wound
