# Supplementary material for: Bead-jet printing enabled sparse mesenchymal stem cell patterning augments skeletal muscle and hair follicle regeneration
Source: Nat Commun. 2022 Dec 3;13:7463. doi: 10.1038/s41467-022-35183-8 (PMC9718784; doi:10.1038/s41467-022-35183-8)
Supplement: Supplementary file 10 — Reporting Summary [file 41467_2022_35183_MOESM10_ESM.pdf]

nature portfolio

Corresponding author(s): Shaohua Ma

Last updated by author(s): 2022/11/11

Reporting Summary

Nature Portfolio wishes to improve the reproducibility of the work that we publish. This form provides structure for consistency and transparency in reporting. For further information on Nature Portfolio policies, see our Editorial Policies and the Editorial Policy Checklist.

Statistics

For all statistical analyses, confirm that the following items are present in the figure legend, table legend, main text, or Methods section.

n/a	Confirmed	
☐	☒	The exact sample size (*n*) for each experimental group/condition, given as a discrete number and unit of measurement
☐	☒	A statement on whether measurements were taken from distinct samples or whether the same sample was measured repeatedly
☐	☒	The statistical test(s) used AND whether they are one- or two-sided<br>*Only common tests should be described solely by name; describe more complex techniques in the Methods section.*
☒	☐	A description of all covariates tested
☐	☒	A description of any assumptions or corrections, such as tests of normality and adjustment for multiple comparisons
☐	☒	A full description of the statistical parameters including central tendency (e.g. means) or other basic estimates (e.g. regression coefficient) AND variation (e.g. standard deviation) or associated estimates of uncertainty (e.g. confidence intervals)
☐	☒	For null hypothesis testing, the test statistic (e.g. *F*, *t*, *r*) with confidence intervals, effect sizes, degrees of freedom and *P* value noted<br>*Give P values as exact values whenever suitable.*
☒	☐	For Bayesian analysis, information on the choice of priors and Markov chain Monte Carlo settings
☒	☐	For hierarchical and complex designs, identification of the appropriate level for tests and full reporting of outcomes
☐	☒	Estimates of effect sizes (e.g. Cohen's *d*, Pearson's *r*), indicating how they were calculated

Our web collection on statistics for biologists contains articles on many of the points above.

Software and code

Policy information about availability of computer code

Data collection	All fluorescent images were collected on the fluorescent microscope (Nikon Elcipse Ts2r) and confocal microscope (Nikon A1);<br>All the optical density (OD) values were measured by Synergy H1 microplate reader (Bio-Teck, Winooski, VT, USA);<br>The rheological property of hydrogels was evaluated by a rotational rheometer (MCR302, Anton Paar, Austria);<br>The granular microgels porosity characterization was conducted by confocal microscope (Nikon A1);<br>For LDH/CyQuant assay, metabolic activity assay, MTT assay and cell migration assay, the OD value was measured by Synergy H1 microplate reader (Bio-Teck, Winooski, VT, USA).<br>Flow cytometry was conduted by flow cytometer (BC16129, Beckman Coulter);<br>Extracellular vesicles were ananylzed by NanoSight NS300 instrument (Malvern) with scientific CMOS sensor and the morphology was characterized by Tecnai G2 Spirit Transmission Electron Microscopy;<br>For histologic evaluation of animal tissue, images were captured on a Leica DM1000 microscope;<br>For immufluorescence evaluation of animal tissue, images were acquired under confocal microscope (Nikon A1);<br>DNA and RNA concentration were determined using the Nanodrop 2000 (Thermo, US);<br>The mRNA sequencing of cells was conducted by Illumina HiSeq;<br>The mRNA sequencing of animals was conducted on BGISEQ500 platform (BGI-Shenzhen, China).
Data analysis	The flow cytometry data were analyzed with FlowJo software (version 10).The western blot results were analyzed using imageJ (version 1.52); NTA data was analyzed by NTA 3.0 (Malvern Instruments); The mRNA sequencing data of cells were sequenced on Illumina HiSeq;The mRNA sequencing data of animals were sequenced on BGISEQ500 platform. Differential expression analysis used the DESeq2 Bioconductor package. All the statistical analysis was performed using Origin 2021b (OriginLab Co, Northampton, MA) and Prism 6 (GraphPad, Inc.) software.

For manuscripts utilizing custom algorithms or software that are central to the research but not yet described in published literature, software must be made available to editors and reviewers. We strongly encourage code deposition in a community repository (e.g. GitHub). See the Nature Portfolio guidelines for submitting code & software for further information.

Data

Policy information about availability of data

All manuscripts must include a data availability statement. This statement should provide the following information, where applicable:

- Accession codes, unique identifiers, or web links for publicly available datasets
- A description of any restrictions on data availability
- For clinical datasets or third party data, please ensure that the statement adheres to our policy

The RNA-seq data described in this article are available at Gene Expression Omnibus (GEO) under accessions GSE214868 and GSE197356. The small RNA-seq data are available under GSE215294. All other data needed to support the conclusions in the paper are presented in the paper and/or the supplementary information. Source data are provided with this paper.

Field-specific reporting

Please select the one below that is the best fit for your research. If you are not sure, read the appropriate sections before making your selection.

☒ Life sciences ☐ Behavioural & social sciences ☐ Ecological, evolutionary & environmental sciences

For a reference copy of the document with all sections, see nature.com/documents/nr-reporting-summary-flat.pdf

Life sciences study design

All studies must disclose on these points even when the disclosure is negative.

Sample size	Sample sizes were chosen based on cell and animal availability or number of experimental or control groups needed to draw conclusions, but were not calculated prior to experiments. The resulting data were sufficient to show significance of reported data based on magnitudes of differences between groups.
Data exclusions	No data acquired for quantitative analysis were excluded.
Replication	All experiments in this study was independently replicated, with a minimum of three biological and technical replicates as indicated in the test or corresponding figure legends.
Randomization	All animals were randomly allocated into each experimental groups for all animal study. All other experiments were conducted with randomly allocated samples and groups.
Blinding	The same investigators both designed and performed experiments and data analysis, therefore no blinding concerning sample identitiy.

Behavioural & social sciences study design

All studies must disclose on these points even when the disclosure is negative.

Study description	*Briefly describe the study type including whether data are quantitative, qualitative, or mixed-methods (e.g. qualitative cross-sectional, quantitative experimental, mixed-methods case study).*
Research sample	*State the research sample (e.g. Harvard university undergraduates, villagers in rural India) and provide relevant demographic information (e.g. age, sex) and indicate whether the sample is representative. Provide a rationale for the study sample chosen. For studies involving existing datasets, please describe the dataset and source.*
Sampling strategy	*Describe the sampling procedure (e.g. random, snowball, stratified, convenience). Describe the statistical methods that were used to predetermine sample size OR if no sample-size calculation was performed, describe how sample sizes were chosen and provide a rationale for why these sample sizes are sufficient. For qualitative data, please indicate whether data saturation was considered, and what criteria were used to decide that no further sampling was needed.*
Data collection	*Provide details about the data collection procedure, including the instruments or devices used to record the data (e.g. pen and paper, computer, eye tracker, video or audio equipment) whether anyone was present besides the participant(s) and the researcher, and whether the researcher was blind to experimental condition and/or the study hypothesis during data collection.*
Timing	*Indicate the start and stop dates of data collection. If there is a gap between collection periods, state the dates for each sample cohort.*
Data exclusions	*If no data were excluded from the analyses, state so OR if data were excluded, provide the exact number of exclusions and the rationale behind them, indicating whether exclusion criteria were pre-established.*
Non-participation	*State how many participants dropped out/declined participation and the reason(s) given OR provide response rate OR state that no participants dropped out/declined participation.*

Randomization	*If participants were not allocated into experimental groups, state so OR describe how participants were allocated to groups, and if allocation was not random, describe how covariates were controlled.*

Ecological, evolutionary & environmental sciences study design

All studies must disclose on these points even when the disclosure is negative.

Study description	*Briefly describe the study. For quantitative data include treatment factors and interactions, design structure (e.g. factorial, nested, hierarchical), nature and number of experimental units and replicates.*
Research sample	*Describe the research sample (e.g. a group of tagged Passer domesticus, all Stenocereus thurberi within Organ Pipe Cactus National Monument), and provide a rationale for the sample choice. When relevant, describe the organism taxa, source, sex, age range and any manipulations. State what population the sample is meant to represent when applicable. For studies involving existing datasets, describe the data and its source.*
Sampling strategy	*Note the sampling procedure. Describe the statistical methods that were used to predetermine sample size OR if no sample-size calculation was performed, describe how sample sizes were chosen and provide a rationale for why these sample sizes are sufficient.*
Data collection	*Describe the data collection procedure, including who recorded the data and how.*
Timing and spatial scale	*Indicate the start and stop dates of data collection, noting the frequency and periodicity of sampling and providing a rationale for these choices. If there is a gap between collection periods, state the dates for each sample cohort. Specify the spatial scale from which the data are taken*
Data exclusions	*If no data were excluded from the analyses, state so OR if data were excluded, describe the exclusions and the rationale behind them, indicating whether exclusion criteria were pre-established.*
Reproducibility	*Describe the measures taken to verify the reproducibility of experimental findings. For each experiment, note whether any attempts to repeat the experiment failed OR state that all attempts to repeat the experiment were successful.*
Randomization	*Describe how samples/organisms/participants were allocated into groups. If allocation was not random, describe how covariates were controlled. If this is not relevant to your study, explain why.*
Blinding	*Describe the extent of blinding used during data acquisition and analysis. If blinding was not possible, describe why OR explain why blinding was not relevant to your study.*

Did the study involve field work? ☐ Yes ☐ No

Field work, collection and transport

Field conditions	*Describe the study conditions for field work, providing relevant parameters (e.g. temperature, rainfall).*
Location	*State the location of the sampling or experiment, providing relevant parameters (e.g. latitude and longitude, elevation, water depth).*
Access & import/export	*Describe the efforts you have made to access habitats and to collect and import/export your samples in a responsible manner and in compliance with local, national and international laws, noting any permits that were obtained (give the name of the issuing authority, the date of issue, and any identifying information).*
Disturbance	*Describe any disturbance caused by the study and how it was minimized.*

Reporting for specific materials, systems and methods

We require information from authors about some types of materials, experimental systems and methods used in many studies. Here, indicate whether each material, system or method listed is relevant to your study. If you are not sure if a list item applies to your research, read the appropriate section before selecting a response.

Materials & experimental systems

n/a	Involved in the study	
☐	☒	Antibodies
☐	☒	Eukaryotic cell lines
☒	☐	Palaeontology and archaeology
☐	☒	Animals and other organisms
☐	☒	Human research participants
☒	☐	Clinical data
☒	☐	Dual use research of concern

Methods

n/a	Involved in the study	
☒	☐	ChIP-seq
☐	☒	Flow cytometry
☒	☐	MRI-based neuroimaging

Antibodies

Antibodies used

Western Blot detection: p-Akt (1:1000, ABclonal, AP0637), Akt (1:1000, ABclonal, A17909), p-GSK3β (1:1000, ABclonal, AP1088), GSK3β (1:1000, ABclonal, A6164), β-CATENIN (1:1000, ABclonal, A19657), β-actin (1:1000, ABclonal, AC026), anti-CD9 (1:1000, Abcam, ab263019), anti-CD81 (1:1000, Abcam, ab109201), anti-Hsp70 (1:1000, Abcam, ab181606), anti-TSG101 (1:1000, Abcam, ab125011), anti-Calnexin (1:1000, Abcam, ab133615), anti-CD63 (1:1000, Abcam, ab134045)

Immunofluorescence: rabbit anti-desmin (1:500, Servicebio, GB11081), rabbit anti-MYH7 (1:400, Servicebio, GB111857), mouse anti-human/mouse/rat/chicken Pax7 (5 μg/ml; R&D Systems, MAB1675), rabbit anti-CD31 (1:200, Servicebio, GB11063-3), rabbit anti-α-SMA (1:100, Servicebio, GB111364), rabbit anti-NF (1:100, Abcam, ab223343), rabbit anti-βIIIT (1:100, Abcam, ab229590), rat anti-CD68 (1:100, Invitrogen, Cat# 14-0681-82), rabbit anti-CD3 (1:100, Abcam, ab16669), rabbit anti-Ki67 (1:600, Servicebio, GB111141), rabbit anti-HLA (1:100, Abcam, ab52922), pAKT (1:100, Cell Signaling, Cat # 4060S), AKT (1:100, Cell Signaling, Cat # 4685S) and β-CATENIN (1:100, Cell Signaling, Cat # 8480S), Alexa Fluor 488 conjugated secondary antibody (1:500, Abcam, Cat # ab150073).

Flow cytometry: phycoerythrin (PE)-conjugated human antibody CD90 (5 μl per million cells in 100 μl staining volume, BioLegend, Cat # 328123, Clone 5E10) and allophycocyanin (APC)-conjugated human antibody CD105 (5 μL per million cells in 100 μL staining volume, BioLegend, Cat # 323225, Clone 43A3)

Validation

All antibodies used are commercially available and have been validated for the application used by the manufacturer.

Western Blot detection:
p-Akt (1:1000, ABclonal, AP0637) https://abclonal.com.cn/catalog/AP0637
Akt (1:1000, ABclonal, A17909) https://abclonal.com.cn/catalog/A17909
p-GSK3β (1:1000, ABclonal, AP1088) https://abclonal.com.cn/catalog/AP1088
GSK3β (1:1000, ABclonal, A6164) https://abclonal.com.cn/catalog/A6164
β-CATENIN (1:1000, ABclonal, A19657) https://abclonal.com.cn/catalog/A19657
β-actin (1:1000, ABclonal, AC026) https://abclonal.com.cn/catalog/A17909
anti-CD9 (1:1000, Abcam, ab263019) https://www.abcam.com/cd9-antibody-epr23105-125-ab263019.html
anti-CD81 (1:1000, Abcam, ab109201) https://www.abcam.com/cd81-antibody-epr4244-ab109201.html
anti-Hsp70 (1:1000, Abcam, ab181606) https://www.abcam.com/hsp70-antibody-epr16892-ab181606.html
anti-TSG101 (1:1000, Abcam, ab125011) https://www.abcam.com/tsg101-antibody-epr7130b-ab125011.html
anti-Calnexin (1:1000, Abcam, ab133615) https://www.abcam.com/calnexin-antibody-epr36332-er-membrane-marker-ab133615.html
anti-CD63 (1:1000, Abcam, ab134045) https://www.abcam.com/cd63-antibody-epr5702-ab134045.html

Immunofluorescence:
rabbit anti-desmin (1:500, Servicebio, GB11081) https://www.servicebio.cn/goodsdetail?id=1364
rabbit anti-MYH7 (1:400, Servicebio, GB111857) https://www.servicebio.cn/goodsdetail?id=4863
mouse anti-human/mouse/rat/chicken Pax7 (5 μg/ml; R&D Systems, MAB1675) https://www.rndsystems.com/cn/products/human-mouse-rat-chicken-pax7-antibody-pax7_mab1675
rabbit anti-CD31 (1:200, Servicebio, GB11063-3) https://www.servicebio.cn/goodsdetail?id=1346
rabbit anti-α-SMA (1:100, Servicebio, GB111364) https://www.servicebio.cn/goodsdetail?id=3743
rabbit anti-NF (1:100, Abcam, ab223343) https://www.abcam.com/68kda-neurofilamentnf-l-antibody-epr22035-112-ab223343.html
rabbit anti-βIIIT (1:100, Abcam, ab229590) https://www.abcam.com/neuron-specific-beta-iii-tubulin-antibody-ab229590.html
rat anti-CD68 (1:100, Invitrogen, Cat# 14-0681-82) https://www.thermofisher.cn/cn/zh/antibody/product/CD68-Antibody-clone-FA-11-Monoclonal/14-0681-82
rabbit anti-CD3 (1:100, Abcam, ab16669) https://www.abcam.com/CD3-antibody-SP7-ab16669.html
rabbit anti-Ki67 (1:600, Servicebio, GB111141), rabbit anti-HLA (1:100, Abcam, ab52922) https://www.abcam.com/hla-a-antibody-ep1395y-ab52922.html
pAKT (1:100, Cell Signaling, Cat # 4060S) https://www.cellsignal.com/products/primary-antibodies/phospho-akt-ser473-d9e-xp-rabbit-mab/4060?site-search-type=Products&N=4294956287&Ntt=4060s&fromPage=plp&_requestid=9826707
AKT (1:100, Cell Signaling, Cat # 4685S) https://www.cellsignal.com/products/primary-antibodies/akt-pan-11e7-rabbit-mab/4685?site-search-type=Products&N=4294956287&Ntt=4685s&fromPage=plp&_requestid=9826894
β-CATENIN (1:100, Cell Signaling, Cat # 8480S) https://www.cellsignal.com/products/primary-antibodies/b-catenin-d10a8-xp-rabbit-mab/8480?site-search-type=Products&N=4294956287&Ntt=8480s&fromPage=plp&_requestid=9826988
Alexa Fluor 488 conjugated secondary antibody (1:500, Abcam, Cat # ab150073) https://www.abcam.com/donkey-rabbit-igg-hl-alexa-fluor-488-ab150073.html .

Flow cytometry:
phycoerythrin (PE)-conjugated human antibody CD90 (5 μl per million cells in 100 μl staining volume, BioLegend, Cat # 328123, Clone 5E10): https://www.biolegend.com/en-us/products/pe-cyanine7-anti-human-cd90-thy1-antibody-8282
allophycocyanin (APC)-conjugated human antibody CD105 (5 μL per million cells in 100 μL staining volume, BioLegend, Cat # 323225, Clone 43A3): https://www.biolegend.com/en-us/products/apc-fire-750-anti-human-cd105-antibody-20089

Eukaryotic cell lines

Policy information about cell lines

Cell line source(s)	Mesenchymal Stem Cells (MSCs) were extracted from human umbilical cord tissues from full-term births after normal vaginal delivery; C2C12 mouse muscle cells (GDC0175), NIH3T3 mouse embryonic fibroblast cells (GDC0030) were purchased from China Center for Type Culture Collection and used in P5 to P20 . SW620 human colon cancer cells (CL-0225) were purchased from Procell Life Science Technology Co,. Ltd.
Authentication	Mesenchymal Stem Cells were analyzed by flow cytometry for MSC markers CD90 and CD105, as suggested by the International Society for Cell Therapies (ISCT). Other cell lines used in this study were authenticated with STR profiling.
Mycoplasma contamination	All cell lines tested negative for mycoplasma.
Commonly misidentified lines (See ICLAC register)	None of the cell lines are listed in the ICLAC database of commonly misidentified cell lines.

Palaeontology and Archaeology

Specimen provenance	*Provide provenance information for specimens and describe permits that were obtained for the work (including the name of the issuing authority, the date of issue, and any identifying information). Permits should encompass collection and, where applicable, export.*
Specimen deposition	*Indicate where the specimens have been deposited to permit free access by other researchers.*
Dating methods	*If new dates are provided, describe how they were obtained (e.g. collection, storage, sample pretreatment and measurement), where they were obtained (i.e. lab name), the calibration program and the protocol for quality assurance OR state that no new dates are provided.*

☐ Tick this box to confirm that the raw and calibrated dates are available in the paper or in Supplementary Information.

Ethics oversight	*Identify the organization(s) that approved or provided guidance on the study protocol, OR state that no ethical approval or guidance was required and explain why not.*

Note that full information on the approval of the study protocol must also be provided in the manuscript.

Animals and other organisms

Policy information about studies involving animals; ARRIVE guidelines recommended for reporting animal research

Laboratory animals	All the animal experiments were performed under the ethical regulation of Shenzhen International Graduate School (SIGS) of Tsinghua University. Mice were housed in a pathogen-free environment with the temperature maintained at 23 ± 2°C and relative humidity at 50 to 65% under a 12 h/12 h light/dark cycle with free access to food and water, in accordance with the National Institutes of Health guidelines. The skeletal muscle defect in GM muscle was created in balb/c mice (male, 4-6 weeks old, Guangdong Medical Laboratory Animal Center, China); The mouse excisional wound-healing model was used in balb/c mice (male, 4-6 weeks old, Guangdong Medical Laboratory Animal Center, China).
Wild animals	None
Field-collected samples	None
Ethics oversight	All the animal experiments were performed under the ethical regulation of Tsinghua University Shenzhen International Graduate School (SIGS) and housed in SIGS Animal Facility in accordance with the National Institutes of Health guidelines

Note that full information on the approval of the study protocol must also be provided in the manuscript.

Human research participants

Policy information about studies involving human research participants

Population characteristics	Human umbilical cord tissues were obtained from full-term births after normal vaginal delivery at the delivery room of the Shenzhen Maternity and Child Healthcare Hospital as approved by the ethics committee. Donors at Shenzhen Maternity and Child Healthcare Hospital volunteered to give the human umbilical cords for this study with informed consent.
Recruitment	Donors were recruited at Shenzhen Maternity and Child Healthcare Hospital with informed consent.
Ethics oversight	All experiments were approved by and performed in accordance with the guidelines of the Shenzhen Maternity and Child Healthcare Hospital Ethics Committee.

Note that full information on the approval of the study protocol must also be provided in the manuscript.

Clinical data

Policy information about clinical studies

All manuscripts should comply with the ICMJE guidelines for publication of clinical research and a completed CONSORT checklist must be included with all submissions.

Clinical trial registration	*Provide the trial registration number from ClinicalTrials.gov or an equivalent agency.*
Study protocol	*Note where the full trial protocol can be accessed OR if not available, explain why.*
Data collection	*Describe the settings and locales of data collection, noting the time periods of recruitment and data collection.*
Outcomes	*Describe how you pre-defined primary and secondary outcome measures and how you assessed these measures.*

Dual use research of concern

Policy information about dual use research of concern

Hazards

Could the accidental, deliberate or reckless misuse of agents or technologies generated in the work, or the application of information presented in the manuscript, pose a threat to:

No	Yes	
☐	☐	Public health
☐	☐	National security
☐	☐	Crops and/or livestock
☐	☐	Ecosystems
☐	☐	Any other significant area

Experiments of concern

Does the work involve any of these experiments of concern:

No	Yes	
☐	☐	Demonstrate how to render a vaccine ineffective
☐	☐	Confer resistance to therapeutically useful antibiotics or antiviral agents
☐	☐	Enhance the virulence of a pathogen or render a nonpathogen virulent
☐	☐	Increase transmissibility of a pathogen
☐	☐	Alter the host range of a pathogen
☐	☐	Enable evasion of diagnostic/detection modalities
☐	☐	Enable the weaponization of a biological agent or toxin
☐	☐	Any other potentially harmful combination of experiments and agents

ChIP-seq

Data deposition

☐ Confirm that both raw and final processed data have been deposited in a public database such as GEO.

☐ Confirm that you have deposited or provided access to graph files (e.g. BED files) for the called peaks.

Data access links<br>*May remain private before publication.*	*For "Initial submission" or "Revised version" documents, provide reviewer access links. For your "Final submission" document, provide a link to the deposited data.*
Files in database submission	*Provide a list of all files available in the database submission.*
Genome browser session<br>(e.g. UCSC)	*Provide a link to an anonymized genome browser session for "Initial submission" and "Revised version" documents only, to enable peer review. Write "no longer applicable" for "Final submission" documents.*

Methodology

Replicates	*Describe the experimental replicates, specifying number, type and replicate agreement.*
Sequencing depth	*Describe the sequencing depth for each experiment, providing the total number of reads, uniquely mapped reads, length of reads and whether they were paired- or single-end.*

Antibodies | *Describe the antibodies used for the ChIP-seq experiments; as applicable, provide supplier name, catalog number, clone name, and lot number.*

Peak calling parameters | *Specify the command line program and parameters used for read mapping and peak calling, including the ChIP, control and index files used.*

Data quality | *Describe the methods used to ensure data quality in full detail, including how many peaks are at FDR 5% and above 5-fold enrichment.*

Software | *Describe the software used to collect and analyze the ChIP-seq data. For custom code that has been deposited into a community repository, provide accession details.*

Flow Cytometry

Plots

Confirm that:

- [x] The axis labels state the marker and fluorochrome used (e.g. CD4-FITC).
- [x] The axis scales are clearly visible. Include numbers along axes only for bottom left plot of group (a 'group' is an analysis of identical markers).
- [x] All plots are contour plots with outliers or pseudocolor plots.
- [x] A numerical value for number of cells or percentage (with statistics) is provided.

Methodology

Sample preparation | MSCs from the third generation were cultured in 2D condition, Matrigel HDG, LDB and HDB groups for 4 days and then harvested in Cell Staining Buffer (420201, BioLegend) and stained with fluorescent-conjugated primary antibodies, phycoerythrin (PE)-conjugated human antibody CD90 (BioLegend) and allophycocyanin (APC)-conjugated human antibody CD105 (BioLegend) for 30 min at 4 °C in the dark, respectively. Processed cells were then washed twice with wash buffer and analyzed by flow cytometry (BC16129, Beckman Coulter).

Instrument | Flow cytometer (BC16129, Beckman Coulter)

Software | FlowJo (version 10, BD Biosciences)

Cell population abundance | No cell sorting was performed.

Gating strategy | All cells were gated on standard FSC vs SSC gating and suitable fluorescence intensity.

- [x] Tick this box to confirm that a figure exemplifying the gating strategy is provided in the Supplementary Information.

Magnetic resonance imaging

Experimental design

Design type | *Indicate task or resting state; event-related or block design.*

Design specifications | *Specify the number of blocks, trials or experimental units per session and/or subject, and specify the length of each trial or block (if trials are blocked) and interval between trials.*

Behavioral performance measures | *State number and/or type of variables recorded (e.g. correct button press, response time) and what statistics were used to establish that the subjects were performing the task as expected (e.g. mean, range, and/or standard deviation across subjects).*

Acquisition

Imaging type(s) | *Specify: functional, structural, diffusion, perfusion.*

Field strength | *Specify in Tesla*

Sequence & imaging parameters | *Specify the pulse sequence type (gradient echo, spin echo, etc.), imaging type (EPI, spiral, etc.), field of view, matrix size, slice thickness, orientation and TE/TR/flip angle.*

Area of acquisition | *State whether a whole brain scan was used OR define the area of acquisition, describing how the region was determined.*

Diffusion MRI | [ ] Used | [ ] Not used

Preprocessing

Preprocessing software	*Provide detail on software version and revision number and on specific parameters (model/functions, brain extraction, segmentation, smoothing kernel size, etc.).*
Normalization	*If data were normalized/standardized, describe the approach(es): specify linear or non-linear and define image types used for transformation OR indicate that data were not normalized and explain rationale for lack of normalization.*
Normalization template	*Describe the template used for normalization/transformation, specifying subject space or group standardized space (e.g. original Talairach, MNI305, ICBM152) OR indicate that the data were not normalized.*
Noise and artifact removal	*Describe your procedure(s) for artifact and structured noise removal, specifying motion parameters, tissue signals and physiological signals (heart rate, respiration).*
Volume censoring	*Define your software and/or method and criteria for volume censoring, and state the extent of such censoring.*

Statistical modeling & inference

Model type and settings	*Specify type (mass univariate, multivariate, RSA, predictive, etc.) and describe essential details of the model at the first and second levels (e.g. fixed, random or mixed effects; drift or auto-correlation).*
Effect(s) tested	*Define precise effect in terms of the task or stimulus conditions instead of psychological concepts and indicate whether ANOVA or factorial designs were used.*

Specify type of analysis: ☐ Whole brain ☐ ROI-based ☐ Both

Statistic type for inference (See Eklund et al. 2016)	*Specify voxel-wise or cluster-wise and report all relevant parameters for cluster-wise methods.*
Correction	*Describe the type of correction and how it is obtained for multiple comparisons (e.g. FWE, FDR, permutation or Monte Carlo).*

Models & analysis

n/a	Involved in the study
☐	☐ Functional and/or effective connectivity
☐	☐ Graph analysis
☐	☐ Multivariate modeling or predictive analysis

Functional and/or effective connectivity	*Report the measures of dependence used and the model details (e.g. Pearson correlation, partial correlation, mutual information).*
Graph analysis	*Report the dependent variable and connectivity measure, specifying weighted graph or binarized graph, subject- or group-level, and the global and/or node summaries used (e.g. clustering coefficient, efficiency, etc.).*
Multivariate modeling and predictive analysis	*Specify independent variables, features extraction and dimension reduction, model, training and evaluation metrics.*
